# Global, regional, and national burden of neonatal sepsis and other neonatal infections attributable to low birth weight: a systematic analysis of deaths, and DALYs with predictions to 2031

**DOI:** 10.3389/fped.2025.1690624

**Published:** 2025-12-18

**Authors:** Xuelei Jia, Xiaoxia Sun, Kaifeng Wei

**Affiliations:** 1 Donghai County Hospital of Traditional Chinese Medicine (Donghai TCM Hospital), Lianyungang, Jiangsu, China; 2 School of Chinese Medicine, Nanjing University of Chinese Medicine, Nanjing, Jiangsu, China

**Keywords:** neonatal sepsis and other neonatal infections, low birth weight, global burden of disease, risk factors, prediction

## Abstract

**Background:**

Low birth weight (LBW) is a leading risk factor for neonatal sepsis and other neonatal infections (NSNIs). However, the temporal and spatial trends in the global burden of NSNIs attributable to LBW have not been comprehensively estimated. This study aims to explore the global burden of NSNIs attributable to LBW from 1990 to 2021 and project future trends for the next decade.

**Methods:**

Based on data from the Global Burden of Disease (GBD) Study 2021, we analyzed the global burden of LBW-related NSNIs by the numbers of deaths and disability-adjusted life years (DALYs), and the corresponding age-standardized mortality rate (ASMR) and age-standardized DALYs rate (ASDR) by gender, Socio-demographic Index (SDI), region and country. Trends were quantified by the estimated annual percentage change, and future projections were made with the Autoregressive Integrated Moving Average (ARIMA) model to 2031.

**Results:**

Globally, there were approximately 136.83 thousand deaths and 12.31 million DALYs from NSNIs caused by LBW in 2021. From 1990 to 2021, the overall ASMR (2.75–2.21) and ASDR (247.28–198.94) per 100,000 population showed a downward trend. The burden was significantly higher in males than in females. Low and low-middle SDI quintiles had the highest burden, inversely related to SDI levels. Geographically, Western Sub-Saharan Africa and South Asia recorded the highest deaths and DALYs cases, while Central Europe and Australasia had the lowest. Projections indicate that by 2031, both ASMR and ASDR will continue to decline, expected to decrease by 11.49% compared to 2021.

**Conclusion:**

Although the global burden of NSNIs attributable to LBW declined between 1990 and 2021, significant disparities persist, particularly among male neonates and in the Low SDI region, reflecting unequal access to healthcare. To mitigate the burden of LBW-related NSNIs, future efforts should prioritize enhancing targeted public health initiatives and healthcare interventions tailored to local contexts, while addressing the deficiency in health resource allocation and socioeconomic inequalities.

## 1 Introduction

Neonatal infections are defined as diseases caused by infections with a variety of pathogenic microorganisms (e.g., bacteria, fungi, or viruses) from birth to 28 days, including neonatal sepsis and other neonatal infections (NSNIs), remain a global health threat (1). According to the Global Burden of Disease (GBD) 2021 data, the rates of age-standardized disability-adjusted life years (DALYs) and mortality for NSNIs averaged 190,657.60 and 2,118.49 per 100,000 population, respectively (2). Neonatal sepsis, a systemic inflammatory response syndrome, is one of the leading causes of neonatal morbidity and mortality (3). In 2021, the estimated average number of neonatal sepsis-related cases worldwide was 5.37 million, with 967,000 deaths (4). Despite advances in medicine, the accurate identification of neonatal sepsis through clinical assessment and laboratory testing persists as a significant challenge. Delayed diagnosis and treatment of neonatal sepsis may lead to serious complications, including organ dysfunction, septic shock and even death (5), while survivors of neonatal sepsis are more likely to develop long-term neurodevelopmental deficits such as cognitive deficits and cerebral palsy (6, 7). Therefore, it is critical to track the burden of NSNIs promptly and to take urgent, targeted efforts to effectively identify and manage controllable risk factors.

As a notable and preventable risk factor, low birth weight (LBW) presents a key threat to neonatal health and survival worldwide (8). Among identified risk factors, including short gestation and air pollution, LBW was the leading contributor to the global age-standardized DALYs rate of neonatal disorders in 2021, with NSNIs accounting for approximately 10% of this LBW-related disease burden (9). The elevated risk of NSNIs in LBW infants may be linked to structural and functional immaturity of neonatal organs, as well as the compromised immune system (10). Hence, addressing LBW is a critical intervention to reduce the burden of NSNIs and improve the quality of life for neonates.

Although extensive epidemiologic and clinical studies have clarified the relationship between LBW and NSNIs, previous relevant studies have focused on specific countries or regions, especially developing countries (11, 12). To date, the global burden and regional spatiotemporal trends of LBW-related NSNIs have not been comprehensively estimated. This study utilized the GBD 2021 dataset to analyze the global burden of NSNIs attributable to LBW from 1990 to 2021 and project temporal trends over the next decade, considering a variety of factors such as global trends, gender, Socio-demographic Index (SDI), national and territorial levels. Our study aimed to provide valuable insights into contemporary public health policies that are committed to reducing the burden of NSNIs attributable to LBW.

## 2 Methods

### 2.1 Data sources

GBD 2021 is a huge multinational cooperation that provides estimates for 371 diseases and injuries, and 88 risk factors in 21 GBD regions, and 204 nations/territories over the period 1990–2021. We selected the following parameters: Risk factor under GBD Estimate; Deaths and DALYs under Measure; number and rate under Metric. Number represents the absolute count of deaths and DALYs in the population, while rate denotes these measures per 100,000 population. LBW was selected as the risk factor, NSNIs as the cause. Data for this study were freely obtained from the GBD website (https://ghdx.healthdata.org/gbd-2021). In GBD 2021, mortality estimates for NSNIs were generated using the Cause of Death Ensemble Modeling (CODEm) platform, while LBW exposure levels were derived from the GBD birthweight database using spatiotemporal Gaussian process regression (ST-GPR). The attributable burden was computed through the comparative risk assessment framework. These standardized modeling tools ensure consistency in estimating NSNIs and LBW across locations and years.

### 2.2 Definition

NSNIs include neonatal systemic bloodstream infections and other neonatal infections not otherwise modeled in the GBD study. According to the International Classification of Diseases, 10th Revision, neonatal sepsis corresponds to codes A40.1, B95.1, P36-P36.9 and P38-P39.9 (13). In the GBD 2021 comparative risk assessment (CRA) framework, low birth weight (LBW; <2,500 g) is treated as the risk factor and NSNIs as the outcome, forming the risk-outcome pair used to estimate LBW-attributable burden. The exposure of interest was full-term LBW, defined as neonates with gestational age ≥37 weeks but weighing <2.5 kg (14). GBD derives the attributable burden from population attributable fractions (PAFs), which integrate the exposure distribution, relative risks (RRs), and the theoretical minimum risk exposure level (TMREL). RRs describing the relationship between LBW and neonatal infection-related mortality or morbidity are obtained from systematic reviews and synthesized using Bayesian meta-regression, while the TMREL for birthweight is defined as 2,500–4,000 g. LBW-attributable deaths and DALYs are then estimated by multiplying the PAF by the total NSNIs burden for each location, age group, sex and year.

DALYs provide an integrative measure of LBW-related NSNIs burden and were calculated as the sum of years of life lost due to premature death and years lived with disability (15). Social and demographic development was assessed using the Socio-demographic Index (SDI), which combines per-capita income, educational attainment, and the fertility rate among women under 25 years (16). Higher SDI reflects greater socio-economic development. In this study, countries and regions were categorized into five SDI quantiles: high SDI, medium (high-medium, medium, low-medium) SDI, and low SDI.

### 2.3 Statistical analysis

Age-standardized mortality rate (ASMR) and age-standardized DALYs rate (ASDR) were calculated in GBD 2021 as weighted sums of age-specific rates using the GBD world standard population, i.e., age-standardized rate (ASR) = *Σ*(a_i_·w_i_)/*Σ*w_i_, where a_i_; is the age-specific rate in age group i and w_i_ is the corresponding standard population weight. All estimates are based on 1,000 posterior draws; for each measure, the mean of the draws was taken as the point estimate and the 2.5th and 97.5th percentiles were used as the lower and upper bounds of the 95% uncertainty interval (UI). The estimated annual percentage change (EAPC) was used to evaluate temporal trends in ASMR and ASDR. For each location, a log-linear regression model was fitted with y = ln(ASR) and x = calendar year (y = α + βx + ε), where β is the annual change in log rate. EAPC was calculated as 100 × [exp(β)−1], and its 95% confidence interval (CI) was derived from the standard error of β. The ASR was considered to upward trend if the values of EAPC and its 95% CI greater than 0. Conversely, those values below 0 were considered as a downward trend. If neither condition is met, it indicates that the trend was considered stable. For choropleth maps, we plotted the posterior mean ASMR or ASDR for each location, and the corresponding 95% UIs were taken from the same set of GBD draws (17).

Additionally, we explored the relationships among ASMR, ASDR and SDI across 21 GBD regions. Locally weighted scatterplot smoothing was used to model the nonlinear trend in the data, and Spearman's rank correlation tests were conducted to quantify the relationship between SDI and disease burden indicators. Based on the ASMR and ASDR, we analyzed current trends and projected the future burden from 2022 to 2031 by the Auto Regressive Integrated Moving Average (ARIMA) Model. Stationarity was checked using differencing and inspection of Autocorrelation function (ACF)/Partial Autocorrelation Function (PACF) plots, and ARIMA (0,2,0) was retained based on residual diagnostics. Exogenous covariates were not included because the analysis followed a univariate forecasting approach consistent with GBD trend extensions. Time-series forecasting was performed using the auto.arima() function from the forecast package in R. Because joinpoint modeling is not included in the standard GBD analytical framework, segmented regression was not performed; instead, short-term fluctuations were described narratively. All analyses were conducted using R software (version 4.2.3) and the Joinpoint Regression Program (version 5.1.0). A *P*-value less than 0.05 was considered statistically significant.

## 3 Results

### 3.1 Global burden and temporal trend in NSNIs

**Figure 1:**
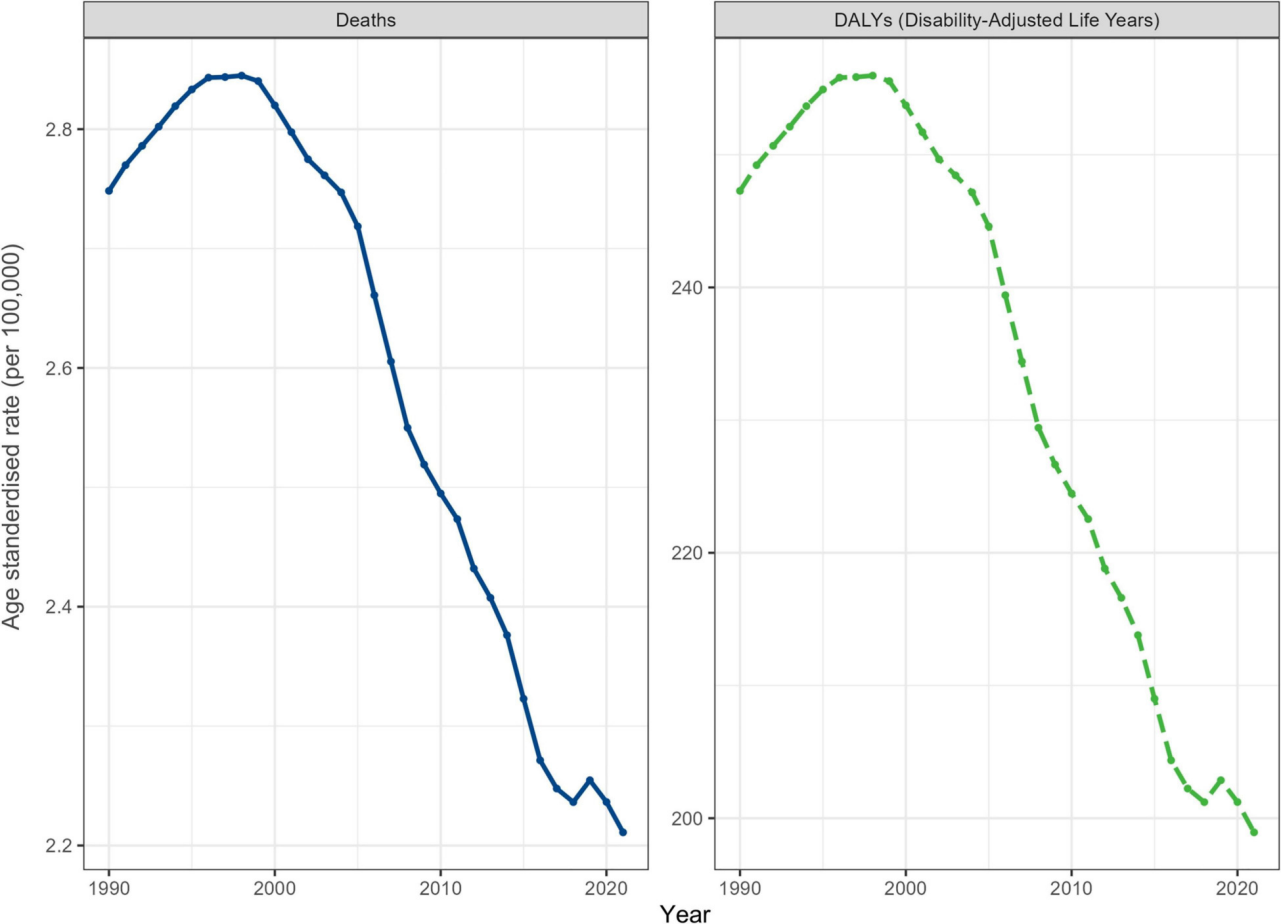
Global trends in age-standardized rates of deaths and disability-adjusted life years for neonatal sepsis and other neonatal infections attributable to low birth weight from 1990 to 2021.

**Figure 2:**
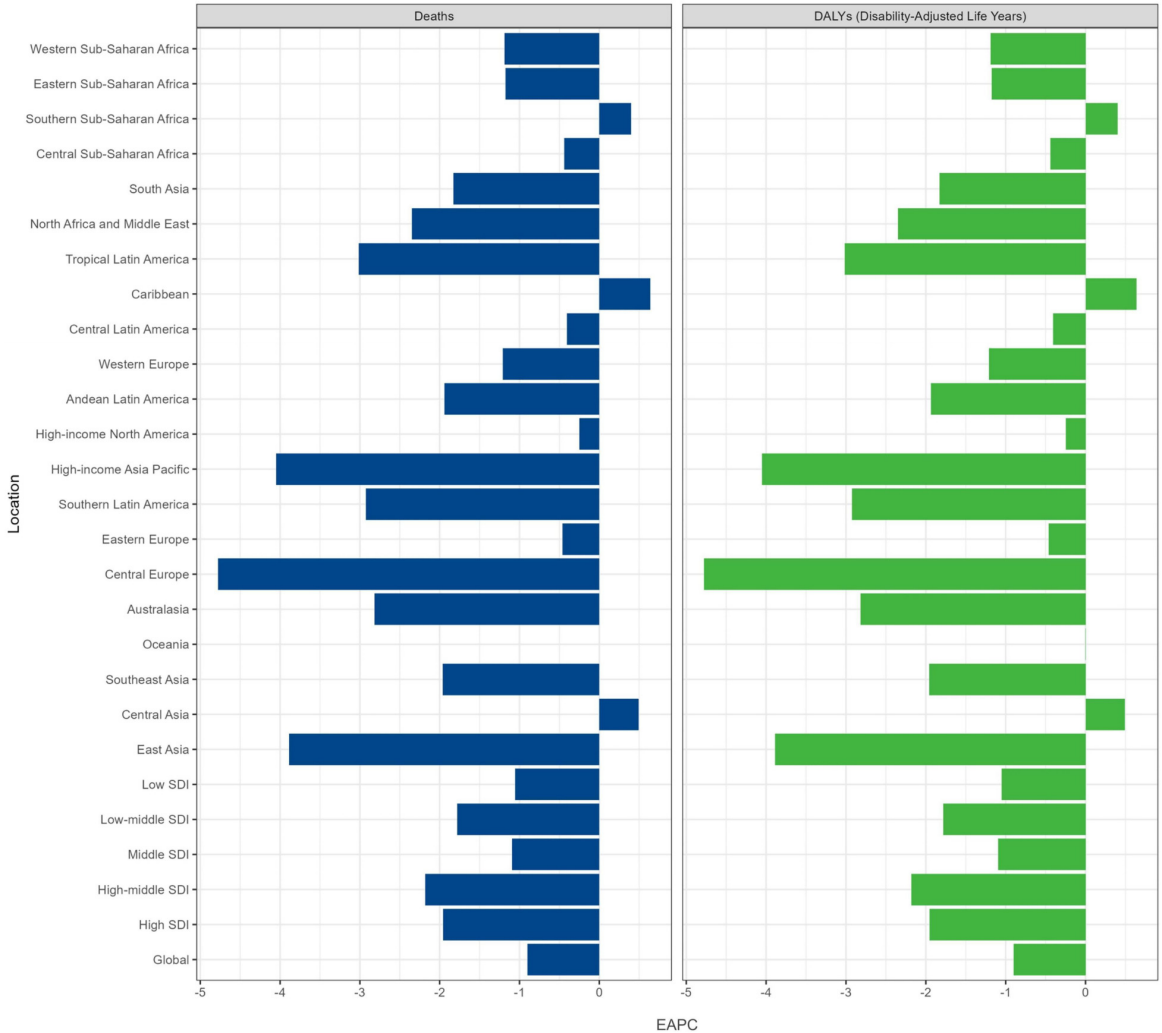
The estimated average percentage change of age-standardized deaths and disability-adjusted life years rate per 100,000 globally from 1990 to 2021.

Globally, the number of deaths decreased by 22.10% from 175,662 [95% UI: 153,836–197,470] in 1990–136,832 (95% UI 116,553–161,001) in 2021 (Figure 1, Table 1). While the corresponding ASMR was 2.75 (95% UI: 2.41–3.09) per 100,000 population in 1990 and 2.21 (1.88–2.60) per 100,000 population in 2021, with the EAPC being −0.90 (95% CI −1.02 to −0.78) (Figure 2). Meanwhile, there were approximately 12.31 million (95% UI 10.49–14.49 million) DALYs caused by LBW-related NSNIs in 2021, which decreased by 22.14% from 15.81 million (95% UI 13.84–17.77) in 1990. From 2019 to 2021, the global ASDR decreased from 247.28 (95% UI 216.58–277.91) to 198.94 (95% UI 169.45–234.07) per 100,000 population (Table 2).

**Table 1:** Numbers and age-standardized rates of deaths for neonatal sepsis and other neonatal infections attributable to low birth weight, with estimated annual percentage change from 1990 to 2021 globally.

Characteristics	Deaths cases			ASMR per 100,000 population		
	1990 No. (95% UI)	2021 No. (95% UI)	EAPC No. (95% CI)	1990 No. (95% UI)	2021 No. (95% UI)	EAPC No. (95% CI)
Global	175,661.81 (153,836.47–197,470.95)	136,831.99 (116,552.68–161,000.74)	−1.96 (−2.08 to −1.84)	2.75 (2.41–3.09)	2.21 (1.88–2.60)	−0.90 (−1.02 to −0.78)
Gender						
Male	103,436.48 (87,957.90–121,812.96)	79,600.58 (66,227.07–96,197.83)	−2.00 (−2.12 to −1.88)	3.13 (2.66–3.68)	2.49 (2.07–3.01)	−0.95 (−1.07 to −0.83)
Female	72,225.33 (63,179.35–81,207.87)	57,231.41 (48,400.57–66,210.80)	−1.89 (−2.03 to −1.76)	2.34 (2.05–2.64)	1.91 (1.62–2.22)	−0.82 (−0.95 to −0.69)
SDI quintile						
High	1,283.32 (1,134.63–1,457.68)	597.89 (528.33–669.31)	−3.01 (−3.2 to −2.83)	0.21 (0.19–0.24)	0.12 (0.11–0.13)	−1.96 (−2.16 to −1.75)
High-middle	4,896.21 (4,244.75–5,624.40)	1,786.01 (1,510.18–2,097.53)	−3.36 (−3.53 to −3.19)	0.56 (0.48–0.64)	0.32 (0.27–0.37)	−2.18 (−2.36 to −2.00)
Middle	29,195.35 (25,378.51–34,019.91)	17,184.94 (14,334.12–20,607.27)	−2.55 (−2.72 to −2.38)	1.46 (1.27–1.70)	1.12 (0.93–1.34)	−1.09 (−1.31 to −0.88)
Low-middle	78,846.32 (67,699.56–90,964.86)	47,192.27 (38,389.38–57,798.24)	−3.23 (−3.34 to −3.12)	4.27 (3.66–4.92)	2.53 (2.06–3.10)	−1.78 (−1.85 to −1.71)
Low	61,335.16 (52,970.60–70,425.31)	69,972.40 (56,244.20–86,912.79)	−2.07 (−2.22 to −1.92)	5.81 (5.03–6.67)	4.06 (3.26–5.04)	−1.05 (−1.11 to −0.99)
GBD region						
Andean Latin America	1,948.62 (1,497.72–2,475.17)	902.12 (660.61–1,212.42)	−3.45 (−3.69 to −3.22)	3.47 (2.66–4.40)	1.51 (1.11–2.04)	−1.94 (−2.15 to −1.73)
Australasia	31.60 (28.02–35.23)	14.10 (11.67–16.95)	−3.49 (−4.26 to −2.71)	0.21 (0.18–0.23)	0.08 (0.07–0.10)	−2.82 (−3.58 to −2.05)
Caribbean	1,334.37 (1,006.70–1,742.33)	1,401.18 (1,004.27–1,873.18)	−0.64 (−0.76 to −0.52)	3.09 (2.33–4.04)	3.67 (2.63–4.91)	0.64 (0.51–0.76)
Central Asia	438.08 (348.61–581.89)	529.89 (436.00–642.09)	0.22 (−0.10–0.54)	0.46 (0.37–0.62)	0.54 (0.44–0.65)	0.49 (0.2–0.79)
Central Europe	137.30 (111.06–166.56)	25.66 (20.54–31.62)	−5.54 (−6.14 to −4.94)	0.17 (0.13–0.20)	0.05 (0.04–0.06)	−4.78 (−5.53 to −4.02)
Central Latin America	3,861.84 (3,557.92–4,228.58)	2,669.50 (2,116.05–3,375.76)	−2.4 (−2.55 to −2.24)	1.61 (1.49–1.77)	1.42 (1.13–1.80)	−0.41 (−0.53 to −0.28)
Central Sub-Saharan Africa	2,584.86 (1,753.88–3,674.20)	3,417.99 (2,025.44–5,680.63)	−1.45 (−1.87 to −1.04)	2.11 (1.43–3.00)	1.60 (0.95–2.66)	−0.44 (−0.71 to −0.17)
East Asia	3,066.63 (2,395.87–3,906.06)	456.36 (341.34–570.00)	−5.61 (−6.15 to −5.07)	0.27 (0.21–0.34)	0.08 (0.06–0.10)	−3.89 (−4.14 to −3.64)
Eastern Europe	629.66 (572.92–699.19)	384.97 (340.88–434.64)	−0.26 (−0.72 to 0.21)	0.44 (0.40–0.49)	0.44 (0.39–0.50)	0.46 (−0.95 to 0.03)
Eastern Sub-Saharan Africa	29,632.59 (25,329.17–34,771.30)	30,096.19 (23,286.85–38,300.02)	−2.39 (−2.57 to −2.21)	6.93 (5.93–8.13)	4.59 (3.55–5.84)	−1.17 (−1.26 to −1.09)
High-income Asia Pacific	190.90 (158.66–231.38)	32.57 (27.61–38.19)	−5.77 (−5.97 to −5.57)	0.20 (0.17–0.24)	0.06 (0.05–0.07)	−4.05 (−4.23 to −3.87)
High-income North America	392.79 (364.76–421.35)	321.13 (284.05–356.51)	−1.33 (−1.72 to −0.95)	0.18 (0.17–0.19)	0.16 (0.15–0.18)	−0.25 (−0.58–0.08)
North Africa and Middle East	4,001.66 (3,197.39–4,867.00)	2,295.18 (1,737.88–2,912.41)	−3.53 (−3.65 to −3.41)	0.76 (0.61–0.93)	0.40 (0.30–0.51)	−2.35 (−2.47 to −2.22)
Oceania	98.41 (62.35–143.05)	189.85 (110.38–304.99)	−0.33 (−0.55 to −0.11)	0.91 (0.58–1.33)	0.92 (0.54–1.49)	0.00 (−0.25–0.25)
South Asia	69,901.99 (59,824.68–83,275.23)	38,422.70 (30,995.10–46,984.10)	−3.70 (−3.8 to −3.59)	4.27 (3.65–5.08)	2.54 (2.05–3.11)	−1.83 (−1.91 to −1.75)
Southeast Asia	18,700.66 (14,676.93–23,776.88)	9,425.28 (7,407.41–12,393.04)	−3.38 (−3.53 to −3.23)	3.16 (2.48–4.02)	1.75 (1.37–2.30)	−1.96 (−2.10 to −1.82)
Southern Latin America	506.21 (434.42–583.70)	164.92 (127.10–214.89)	−4.35 (−4.61 to −4.08)	1.00 (0.86–1.15)	0.44 (0.34–0.58)	−2.92 (−3.21 to −2.64)
Southern Sub-Saharan Africa	1,769.50 (1,374.80–2,145.74)	1,854.42 (1,460.49–2,316.93)	−0.63 (−0.92 to −0.35)	2.28 (1.77–2.77)	2.37 (1.87–2.97)	0.4 (0.22–0.58)
Tropical Latin America	4,647.14 (4,121.60–5,264.21)	1,731.46 (1,363.38–2,202.83)	−4.17 (−4.61 to −3.73)	2.89 (2.56–3.27)	1.04 (0.82–1.33)	−3.02 (−3.45 to −2.58)
Western Europe	371.53 (340.88–399.76)	201.23 (169.94–235.25)	−1.77 (−2 to −1.53)	0.17 (0.15–0.18)	0.10 (0.09–0.12)	−1.21 (−1.41 to −1.01)
Western Sub-Saharan Africa	31,415.47 (25,786.99–36,668.17)	42,295.30 (35,048.28–50,727.12)	−1.88 (−2.03 to −1.72)	7.38 (6.06–8.62)	4.98 (4.12–5.97)	−1.19 (−1.26 to −1.12)

EAPC, estimated annual percentage change; ASMR, age-standardized mortality rate; UI, uncertainty interval; CI, confidence interval; GBD, global burden of disease; SDI, socio-demographic index.

**Table 2:** Numbers and age-standardized rates of disability-adjusted life years for neonatal sepsis and other neonatal infections attributable to low birth weight, with estimated annual percentage change from 1990 to 2021 globally.

Characteristics	DALYs cases			ASDR per 100,000 population		
	1990 No. (95% UI)	2021 No. (95% UI)	EAPC No. (95% CI)	1990 No. (95% UI)	2021 No. (95% UI)	EAPC No. (95% CI)
Global	15,805,094.48 (13,841,362.63–17,767,380.33)	12,311,418.05 (10,486,796.35–14,485,991.51)	−1.96 (−2.08 to −1.84)	247.28 (216.58–277.91)	198.94 (169.45–234.07)	−0.90 (−1.02 to −0.78)
Gender						
Male	9,306,657.84 (7,913,980.99–10,960,093.27)	7,162,042.37 (5,958,765.16–8,655,372.56)	−2.00 (−2.12 to −1.88)	281.19 (239.08–330.98)	223.84 (186.24–270.52)	−0.95 (−1.07 to −0.83)
Female	6,49,8436.64 (5,684,525.76–7,306,629.92)	5,149,375.69 (4,354,824.61–5,957,297.99)	−1.89 (−2.03 to −1.76)	210.85 (184.46–237.10)	172.26 (199.30–145.68)	−0.82 (−0.95 to −0.69)
SDI quintile						
High	115,466.27 (102,087.35–131,154.14)	53,794.31 (47,536.17–60,220.45)	−3.01 (−3.2 to −2.83)	19.15 (16.92–21.75)	10.82 (9.56–12.12)	−1.96 (−2.16 to −1.75)
High-middle	440,534.14 (381,918.23–506,052.70)	160,695.13 (135,877.87–188,724.09)	−3.36 (−3.53 to −3.19)	50.10 (43.43–57.56)	28.44 (24.05–33.41)	−2.18 (−2.36 to −2.00)
Middle	2,626,838.12 (2,283,419.20–3,060,924.98)	1,546,208.59 (1,289,705.89–1,854,133.16)	−2.55 (−2.72 to −2.38)	131.23 (114.08–152.92)	100.81 (84.07–120.91)	−1.09 (−1.31 to −0.88)
Low-middle	7,094,166.22 (6,091,240.17–8,184,531.89)	4,246,110.63 (3,454,071.96–5,200,381.93)	−3.23 (−3.34 to −3.12)	383.79 (329.55–442.43)	227.69 (185.22–278.85)	−1.78 (−1.85 to −1.71)
Low	5,518,601.81 (4,766,002.87–6,336,496.93)	6,295,747.98 (5,060,556.47–7,819,954.48)	−2.07 (−2.22 to −1.92)	523.18 (452.17–599.97)	365.02 (293.41–453.39)	−1.05 (−1.11 to −0.99)
GBD region						
Andean Latin America	175,326.30 (134,756.87–222,702.95)	81,167.86 (59,438.33–109,087.13)	−3.45 (−3.69 to −3.22)	311.95 (239.77–396.19)	136.31 (99.81–183.19)	−1.94 (−2.15 to −1.73)
Australasia	2,843.65 (2,521.27–3,169.88)	1,268.86 (1,049.74–1,524.67)	−3.49 (−4.26 to −2.71)	18.60 (16.49–20.73)	7.35 (6.08–8.83)	−2.82 (−3.58 to −2.05)
Caribbean	120,059.74 (90,577.80–156,765.19)	126,070.68 (90,358.71–168,538.54)	−0.64 (−0.76 to −0.52)	278.28 (209.95–363.32)	330.53 (236.90–441.87)	0.64 (0.51–0.76)
Central Asia	39,415.87 (31,366.45–52,355.22)	47,676.17 (39,228.64–57,771.28)	0.22 (−0.1–0.54)	41.78 (33.25–55.50)	48.44 (39.85–58.70)	0.49 (0.2–0.79)
Central Europe	12,353.62 (9,992.15–14,986.40)	2,308.47 (1,847.62–2,845.01)	−5.54 (−6.14 to −4.94)	14.92 (12.06–18.10)	4.56 (3.65–5.63)	−5.54 (−6.14 to −4.94)
Central Latin America	347,467.27 (320,121.69–380,463.92)	240,186.80 (190,389.85–303,732.01)	−2.40 (−2.55 to −2.24)	145.08 (133.66–158.86)	127.82 (101.32–161.64)	−2.40 (−2.55 to −2.24)
Central Sub-Saharan Africa	232,571.84 (157,804.78–330,584.82)	307,532.11 (182,237.93–511,112.93)	−1.45 (−1.87 to −1.04)	189.73 (128.77–269.51)	144.00 (85.33–239.30)	−1.45 (−1.87 to −1.04)
East Asia	275,918.59 (215,566.70–351,446.70)	41,060.85 (30,712.04–51,285.24)	−5.61 (−6.15 to −5.07)	24.03 (18.77–30.62)	7.40 (5.54–9.24)	−5.61 (−6.15 to −5.07)
Eastern Europe	56,652.89 (51,548.08–62,909.46)	34,637.51 (30,670.57–39,106.78)	−0.26 (−0.72 to 0.21)	39.35 (35.80–43.70)	39.99 (35.40–45.16)	0.46 (−0.95 to 0.03)
Eastern Sub-Saharan Africa	2,666,177.83 (2,278,979.13–3,128,533.01)	2,707,896.64 (2,095,227.25–3,446,034.05)	−2.39 (−2.57 to −2.21)	623.60 (533.19–731.34)	413.18 (319.72–525.82)	−1.17 (−1.26 to −1.09)
High-income Asia Pacific	17,175.77 (14,275.43–20,818.07)	2,930.63 (2,484.45–3,436.33)	−5.77 (−5.97 to −5.57)	18.02 (14.98–21.83)	5.10 (4.33–5.99)	−4.05 (−4.23 to −3.87)
High-income North America	35,341.08 (32,819.20–37,910.48)	28,893.59 (25,557.41–32,076.78)	−1.33(−1.72 to −0.95)	16.04 (14.89–17.21)	14.76 (13.05–16.38)	−0.25 (−0.58–0.08)
North Africa and Middle East	360,046.83 (287,682.48–437,904.47)	206,507.46 (156,364.42–262,042.19)	−3.53 (−3.65 to −3.41)	68.77 (54.96–83.65)	35.99 (27.25–45.67)	−2.35 (−2.47 to −2.22)
Oceania	8,854.27 (5,609.71–12,871.17)	17,081.82 (9,931.14–27,441.37)	−0.33 (−0.55 to −0.11)	82.20 (52.08–119.47)	83.22 (48.39–133.69)	0.00 (−0.25–0.25)
South Asia	6,289,408.18 (5,382,708.47–7,492,674.72)	3,457,071.86 (2,788,774.80–4,227,381.70)	−3.7 (−3.8 to −3.59)	383.79 (328.50–456.68)	228.54 (184.37–279.47)	−1.83 (−1.91 to −1.75)
Southeast Asia	1,682,583.83 (1,320,550.74–2,139,314.05)	848,035.99 (666,479.70–1,115,060.70)	−3.38 (−3.53 to −3.23)	284.35 (223.17–361.55)	157.04 (123.42–206.51)	−1.96 (−2.1 to −1.82)
Southern Latin America	45,545.52 (39,086.69–52,518.44)	14,838.28 (11,436.08–19,334.54)	−4.35 (−4.61 to −4.08)	89.73 (77–103.47)	39.73 (30.61–51.77)	−2.92 (−3.21 to −2.64)
Southern Sub-Saharan Africa	159,209.86 (123,696.35–193,062.03)	166,850.81 (131,406.55–208,465.27)	−0.63 (−0.92 to −0.35)	205.37 (159.58–249.02)	213.54 (168.18–266.79)	0.40 (0.22–0.58)
Tropical Latin America	418,122.83 (370,837.34–473,643.47)	155,787.17 (122,669.30–198,198.56)	−4.17 (−4.61 to −3.73)	259.64 (230.28–294.12)	93.96 (73.99–119.53)	−3.02 (−3.45 to −2.58)
Western Europe	33,427.85 (30,670.56–35,968.05)	18,105.60 (15,290.27–21,166.87)	−1.77 (−2 to −1.53)	14.98 (13.74–16.12)	9.15 (7.73–10.7)	−1.21 (−1.41 to −1.01)
Western Sub-Saharan Africa	2,826,590.85 (2,320,169.64–3,299,195.73)	3,805,508.90 (3,153,462.33–4,564,161.70)	−1.88 (−2.03 to −1.72)	663.71 (545.10–775.34)	447.72 (370.93–536.92)	−1.19 (−1.26 to −1.12)

EAPC, estimated annual percentage change; DALYs, disability-adjusted life years; ASDR, age-standardized DALYs rate; UI, uncertainty interval; CI, confidence interval; GBD, global burden of disease; SDI, socio-demographic index.

### 3.2 Variation in LBW-related NSNIs burden by gender

**Figure 3:**
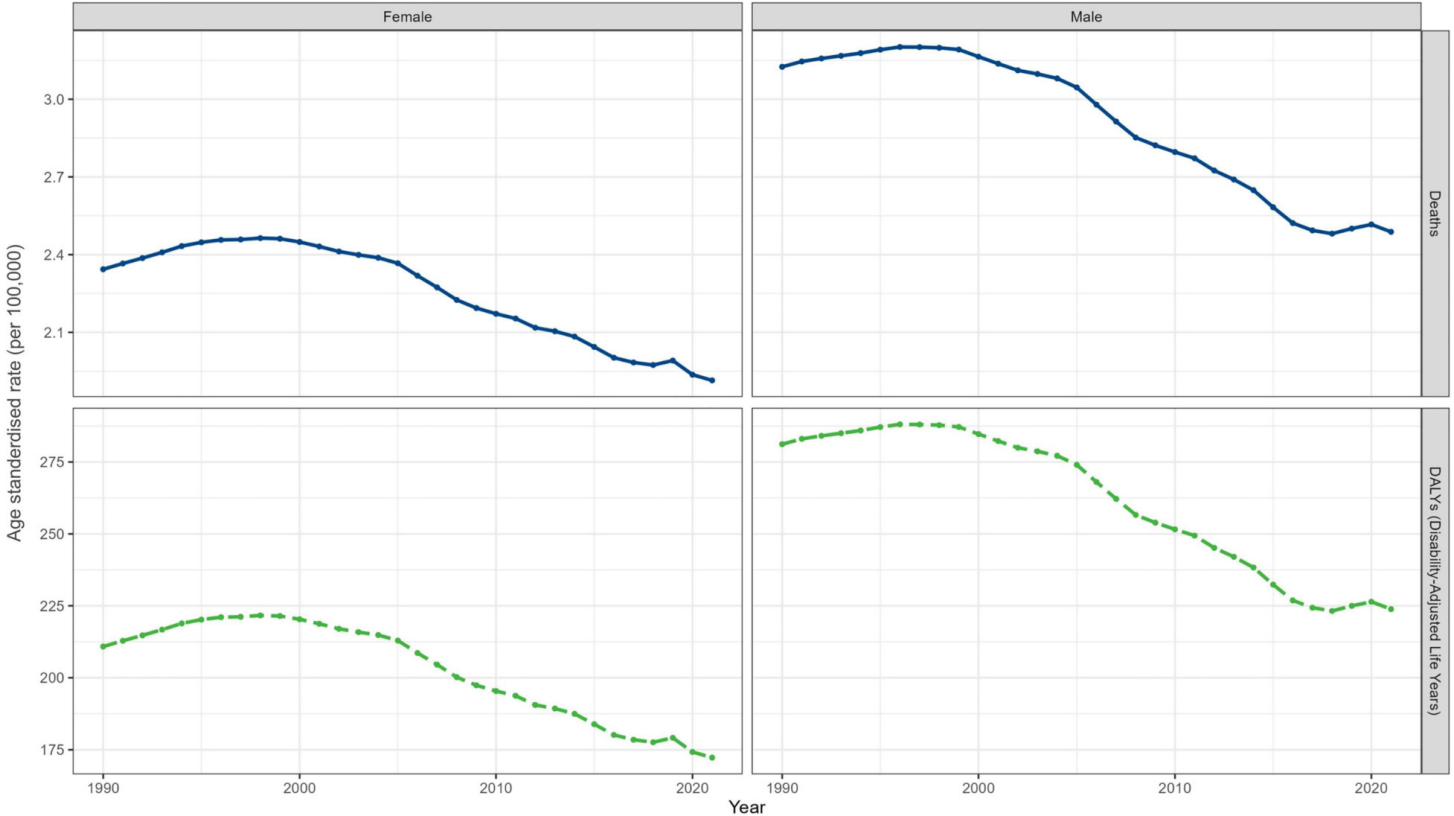
Global trends in age-standardized rates of deaths and disability-adjusted life years from 1990 to 2021, classified by gender.

In 2021, the global number and ASR of deaths and DALYs were higher in males than in females (Tables 1, 2, Figure 3). And the ASMR per 100,000 population [2.49 (95% UI: 2.07–3.01) in males vs. 1.91 (95% UI: 1.62–2.22) in females] and ASDR per 100,000 population [223.84 (95% UI: 186.24–270.52) in males vs. 172.26 (95% UI: 199.30–145.68) in females] were higher in males than in females. From 1990 to 2021, the number of deaths and DALYs for both sexes showed a decreasing trend at the overall level, and the ASMR and ASDR also decreased in males [EAPC = −0.95 (95% CI −1.07 to −0.83)] and females [EAPC = −0.82 (95% CI −0.95 to −0.69)].

### 3.3 Variation in LBW-related NSNIs burden by SDI

**Figure 4:**
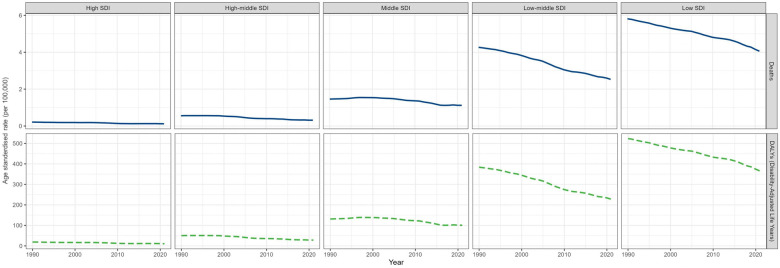
Global trends in age-standardized rates of deaths and disability-adjusted life years from 1990 to 2021, stratified by socio-demographic Index quintiles.

In 2021, lower SDI was associated with higher ASMR and ASDR, with values above the global rate in the low and low-middle SDI quintiles and below the worldwide rate of the other three SDI quintiles (Tables 1, 2, Figure 4). And the highest numbers of deaths and DALYs were observed in the Low SDI quintile [deaths cases 69,972.40 (95% UI: 56,244.20–86,912.79); DALYs cases 6,295,747.98 (95% UI: 5,060,556.47–7,819,954.48)]. From 1990 to 2021, ASMR and ASDR declined in all five SDI quintiles, with the lowest values in the high-middle SDI quintile [EAPC = 95% CI −2.18 (−2.36 to −2.00)] (Figure 2).

### 3.4 Variation in LBW-related NSNIs burden at the regional level

**Figure 5:**
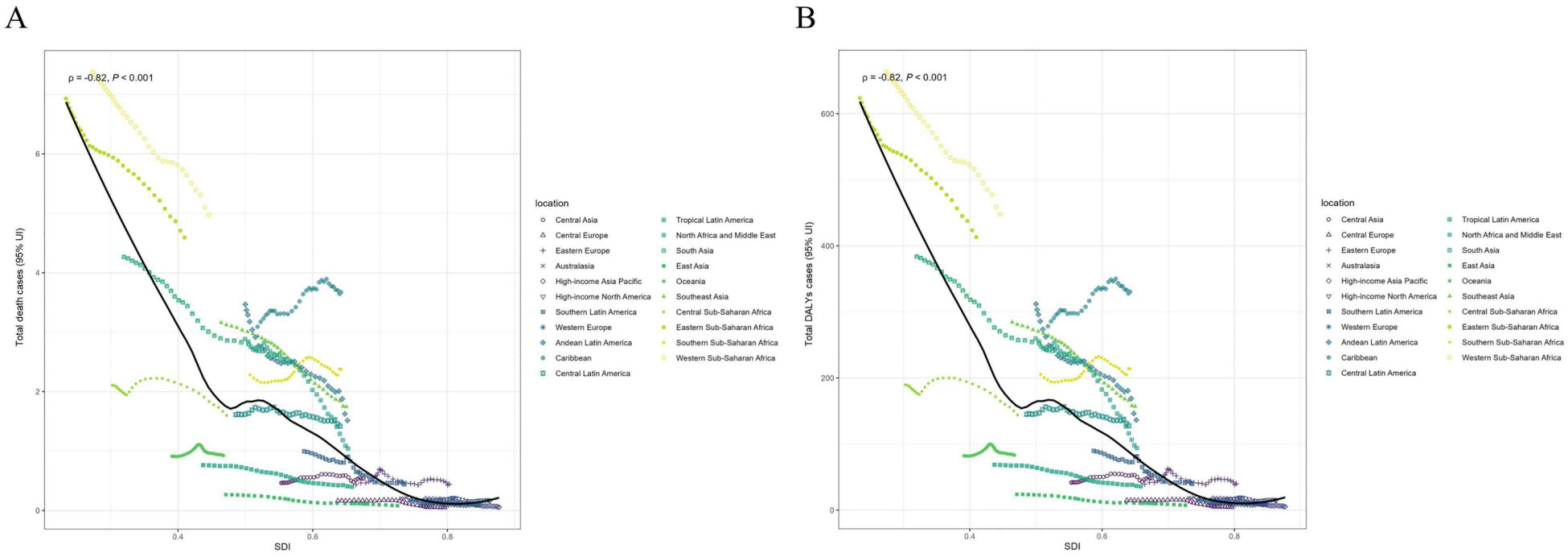
Total deaths cases **(A)** and DALYs cases **(B)** of neonatal sepsis and other neonatal infections attributable to low birth weight across 21 GBD regions by SDI from 1990 to 2021. The black line illustrates the age-standardized rate expected for 2021 based exclusively on the SDI, while the points progressing from left to right represent the estimated values for each region from 1990 to 2021. DALYs, disability-adjusted life years; GBD, Global Burden of Disease; SDI, Socio-demographic Index; UI, uncertainty interval.

Regarding GBD regions, Western Sub-Saharan Africa, South Asia, and Eastern Sub-Saharan Africa were among the top three regions for the highest numbers of deaths and DALYs in 2021, while Australasia, Central Europe, and High-income Asia Pacific had the lowest numbers (Tables 1, 2). Western Sub-Saharan Africa was the region with the highest ASMR [4.98 (95% UI 4.12–5.97)], and ASDR [447.72 (95% UI 370.93–536.92)] per 100,000 in 2021. Central Europe had the lowest ASMR [0.05 (95% UI 0.04–0.06)] and ASDR [4.56 (95% UI 3.65–5.63)] per 100,000 in 2021. The EAPCs of ASMR and ASDR were highest in Caribbean, Central Asia, and Southern Sub-Saharan Africa (EAPC = 0.64, 0.49, and 0.40, respectively) and lowest in Central Europe, High-income Asia Pacific, and East Asia (EAPC = −4.78, −4.05, and −3.89, respectively) from 1990 to 2021 (Figure 2). In addition, 76.19% of the GBD regions experienced a decline in the ASMR and ASDR over 31 years. With the exception of Central Asia, the number of deaths and DALYs decreased in other GBD regions from 1990 to 2021. Figure 5 shows the observed regional ASMR and ASDR associated with the SDI, as well as the expected levels for each location based on the SDI, expressed as an annual time series from 1990 to 2021. Except for Central Asia, the ASMR and ASDR decreased with increasing SDI values in most GBD regions.

### 3.5 Variation in LBW-related NSNIs burden at the national and territorial level

**Figure 6:**
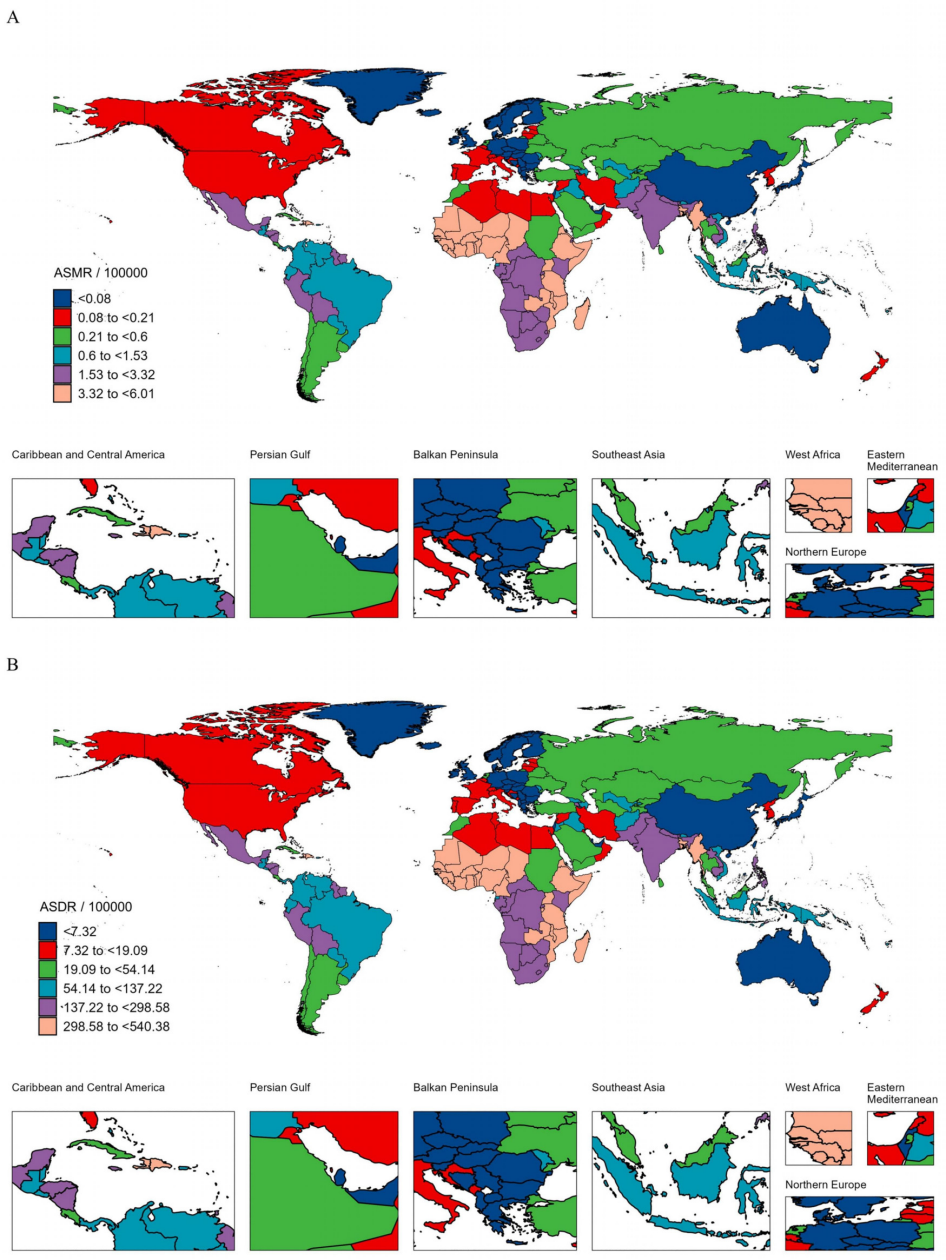
The ASMR **(A)** and ASDR **(B)** per 100,000 of neonatal sepsis and other neonatal infections attributable to low birth weight across countries and territories in 2021. ASMR, age-standardized mortality rate; ASDR, age-standardized disability-adjusted life years rate.

The GBD burden of NSNIs attributable to LBW varied considerably across the world in 2021, with the top three numbers of deaths and DALYs observed in India (23,821.34 deaths and 2.14 million DALYs), Nigeria (20,102.69 deaths and 1.81 million DALYs), and Pakistan (8,770.76 deaths and 0.79 million DALYs). Somalia had the highest ASMR [6.01 (95% UI: 2.99–9.72) per 100,000] and ASDR [540.38 (95% UI: 269.43–874.44) per 100,000, respectively] in 2021, followed by Sierra Leone and Burkina Faso (Figure 6, Supplementary Tables S1, S2). In contrast, the lowest ASMR and ASDR were found in Slovenia [0.0095 (95% UI: 0.0059–0.0138), 0.85 (95% UI: 0.53–1.25), respectively]. As for the absolute number, the lowest number of deaths and DALYs cases was both observed in Andorra (0.01 deaths and 0.55 DALYs). Moreover, the ASR of deaths and DALYs decreased in more than 80% countries or territories between 1990 and 2021. Taiwan (Province of China), Guam, and Georgia ranked in the top three countries or territories experienced a significant increase in the ASMR and ASDR (EAPC = 10.55, 7.16, and 3.32, respectively), while Poland, Saudi Arabia, and Paraguay ranked in the top three countries or territories experienced a significant decrease (EAPC = −7.92, −5.36, and −5.21, respectively).

### 3.6 Predictive analysis for the next decade

Informed by the ACF and PACF plots, a battery of ARIMA (p, d, q) models were evaluated to identify the optimal fit for ARMS and ASDR (Supplementary Figure S1). After filtering by the auto.arima() function, the optimized models for the ASMR and ASDR were both (0,2,0), with AIC values of −165.31 and 104.66, BIC values of −163.91 and 106.06, respectively. The observed and fitted values were in good agreement (cor = 0.998, *P* ≤ 0001). The residuals were normally distributed according to the *Q*-*Q*, ACF, and PACF plots, and the Ljung-Box test confirmed that the residuals of the model were white noise (*Q* = 7.835, *P* = 0.25).

**Figure 7:**
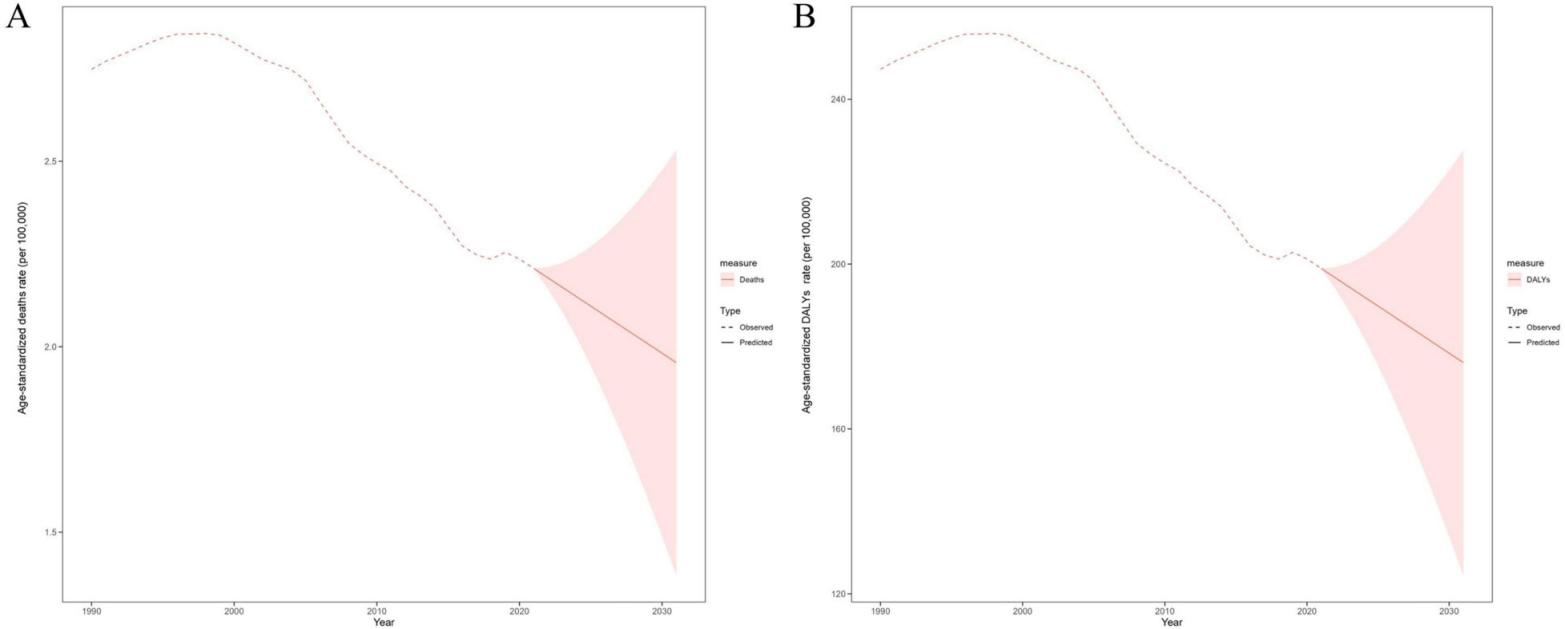
Predicted the global trends of age-standardized deaths rate **(A)** and age-standardized DALYs rate **(B)** per 100,000 for neonatal sepsis and other neonatal infections attributable to low birth weight over the next decade. The red dotted line represents the observed trend between 1990 and 2021; the red line and shaded area represent the predicted trend from 2022 to 2031 and its 95% confidence interval, respectively. DALYs, disability-adjusted life years.

According to the prediction results suggest that if unmitigated, the ASMR and ASDR in 2031 both decreased by 11.49% compared with those in 2021 to 1.96 (95% UI: 1.38–2.53) and 176.08 (95% UI: 124.53–227.64) per 100,000 population, respectively (Figure 7, Supplementary Table S3).

## 4 Discussion

This study utilized data from the GBD study to comprehensively assess the global burden of NSNIs attributable to LBW between 1990 and 2021. It also projects trends from 2022 to 2031. In 2021, LBW remained a significant contributor to the global burden of NSNIs, with considerable variation among gender, SDI, GBD region, and country. From 2019 to 2021, From 2019 to 2021, global ASMR and ASDR showed an improving trend, with the burden on males consistently higher than that on females. However, the number of deaths and DALYs for LBW-related NSNIs remained high, particularly in low and low-middle SDI quintiles, such as South Asia, Western and Eastern Sub-Saharan Africa, which underscored the persistent disparities in burden between high- and low-SDI regions. Our projections indicate that the global burden of LBW-related NSNIs will continue to decrease over the next decade. To further mitigate this burden, particularly among males and in low-SDI regions, targeted strategies are crucial.

The World Health Assembly has identified LBW as a core indicator for the assessment of maternal and child health (18). NSNIs associated with LBW has become a major medical challenge in the field of neonatal critical care worldwide (19). Our research has indicated a significant decline in LBW-related NSNIs between 1990 and 2021, which may be closely linked to improvements in global healthcare conditions and preventive management strategies.

Clinical observations have shown that in neonatal sepsis, male newborns often experience severe progression of disease (e.g., neonatal sepsis) and face a poor prognosis (20). Gender disparities were also evident in our study, with males experiencing a significantly higher burden of NSNIs attributable to LBW than females, which may be influenced by a combination of biological and social factors (21–23). Male are more sensitive to unfavorable perinatal and postnatal environmental conditions and are prone to LBW, which may further increase the risk of NSNIs (24). Gender differences are associated with the expression of sex hormones, sex chromosomes, and sex-specific genes from autosomes (25). Certain genetic factors (especially genes located on the *X* chromosome) may contribute to the increased susceptibility of males to neonatal sepsis (26). Over the study period, males consistently showed higher ASMR and ASDR than females, with the absolute difference widening modestly and the relative difference remaining approximately 1.2–1.4-fold; these sex differences may reflect biological susceptibility, immunological immaturity, and sex-related variation in exposure to perinatal risks (27). Additionally, in many Asian countries such as India and China, patriarchal societal attitudes and behaviors that favor boys over girls may influence gender disparities (28). During the allocation of medical resources, male infants often receive greater attention and preferential treatment, which may also contribute to obtaining data on male births more comprehensive (29).

SDI is widely regarded as a pivotal metric for assessing a nation's developmental status. It comprehensively reflects disparities encountered by countries and regions within the health sector (30). Nearly half of all deaths in countries with high neonatal mortality are due to sepsis, making neonatal sepsis one of the leading infectious causes of mortality in low- and middle-income countries (31, 32). Our study observed that the numbers of deaths and DALYs from NSNIs attributable to LBW were significantly and negatively correlated with the level of SDI in 2021. From 1990 to 2021, regions with high and high-middle SDI experienced the fastest declines in ASMR and ASDR, while the Low SDI region declined the slowest and had the highest number of deaths and DALYs, underscoring the persistent health disparities across socioeconomic status. Moreover, there were also significant differences in the geographic distribution of this burden. Our study highlights that South Asia, Western and Eastern Sub-Saharan Africa continue to be significantly impacted by LBW-related NSNIs, while the high-income Asia-Pacific region has the lowest burden. India and Somalia were two countries with the highest number of deaths and DALYs in 2021, respectively. These results all reflect the underlying politics, poverty, health inequalities and health system challenges in less developed regions. Current evidence has proven that sub-Saharan Africa is one of the regions with the highest neonatal mortality due to neonatal sepsis, but until now treatment and prevention strategies in this region have remained unsatisfactory (33). In sub-Saharan Africa and some South Asian countries, there is a shortage of skilled health-care workers and effective obstetric care, with only half of newborns receiving skilled medical delivery services (34, 35). Meanwhile, low economic levels mean that many families cannot access adequate nutrition and health support, particularly in developing countries where maternal malnutrition is a major determinant of LBW in newborns (36). LBW resulting from maternal and infant malnutrition may compromise the immune system function of newborns and increase the risks of infection, underscoring the urgent need to break the intergenerational cycle of ill health and malnutrition in low- and middle-income countries (37). Furthermore, challenges are presented by limited access to vaccination and antibiotic management due to resource constraints. In lower-middle-income countries, the increasing resistance to World Health Organization-recommended therapies for neonatal sepsis undermines the efficacy of sepsis (38). Consequently, addressing these challenges requires not only raising national health awareness, but also collaborative efforts by policymakers, health-care administrators, clinicians and researchers to reduce the impact of NSNIs by increasing the recruitment, training and education of the health workforce, as well as improving health facilities and incentives to work.

The ARIMA model predicts a downward trend in the ASMR and ASDR of NSNIs attributable to LBW by 2031. This emphasizes that while the current burden of LBW-related NSNIs remains high in less developed countries, the overall burden has improved. This may be attributed to initial strengthening of global health initiatives and healthcare services (39). Moving forward, government agencies in low- and middle-income regions should continue to effectively enhance access to neonatal health education while closely monitoring neonatal healthcare efforts.

This study has several limitations. First, this analysis utilized aggregated secondary data in the GBD 2021 database, and the accuracy of these estimates remain insufficient. Second, this study is limited by the availability of data, particularly in low- and middle-income countries. Incomplete or unreliable vital registration systems may affect the representativeness of data, potentially introducing bias into estimates. Thirdly, our study did not distinguish between early-onset and late-onset neonatal sepsis, which limited our ability to assess the impact of onset time on the burden of NSNIs. More detailed clinical data are needed for further research to enhance the precision of burden estimates and provide more valuable information for the management of neonatal infections worldwide.

## 5 Conclusion

This study provides a comprehensive overview of global trends and future projections for NSNIs attributable to LBW. Despite the overall global downward trend, LBW-related NSNIs remain a major global public health challenge, particularly affecting male neonates and low-SDI regions (e.g., South Asia and Sub-Saharan Africa). To effectively address these disparities, future efforts should prioritize enhancing targeted public health initiatives and healthcare interventions tailored to local contexts for these high-burden populations and regions. Addressing the deficiency in health resource allocation and socioeconomic inequalities is also crucial for mitigating the future burden of LBW-related NSNIs.

## Data Availability

The original contributions presented in the study are included in the article/Supplementary Material, further inquiries can be directed to the corresponding authors.
