# Supplementary material for: Global, regional, and national burden of neonatal sepsis and other neonatal infections attributable to low birth weight: a systematic analysis of deaths, and DALYs with predictions to 2031
Source: Front Pediatr. 2025 Dec 18;13:1690624. doi: 10.3389/fped.2025.1690624 (PMC12756400; doi:10.3389/fped.2025.1690624)
Supplement: Supplementary file 1 [file Supplementaryfile1.docx]

Supplementary Material

Global, regional, and national burden of neonatal sepsis and other neonatal infections attributable to low birth weight: a systematic analysis of deaths, and DALYs with predictions to 2031

[Supplementary Figure S1. 2](#_Toc206712085)

[Supplementary Table S1. 3](#_Toc206712086)

[Supplementary Table S2. 10](#_Toc206712087)

[Supplementary Table S3. 21](#_Toc206712088)

**
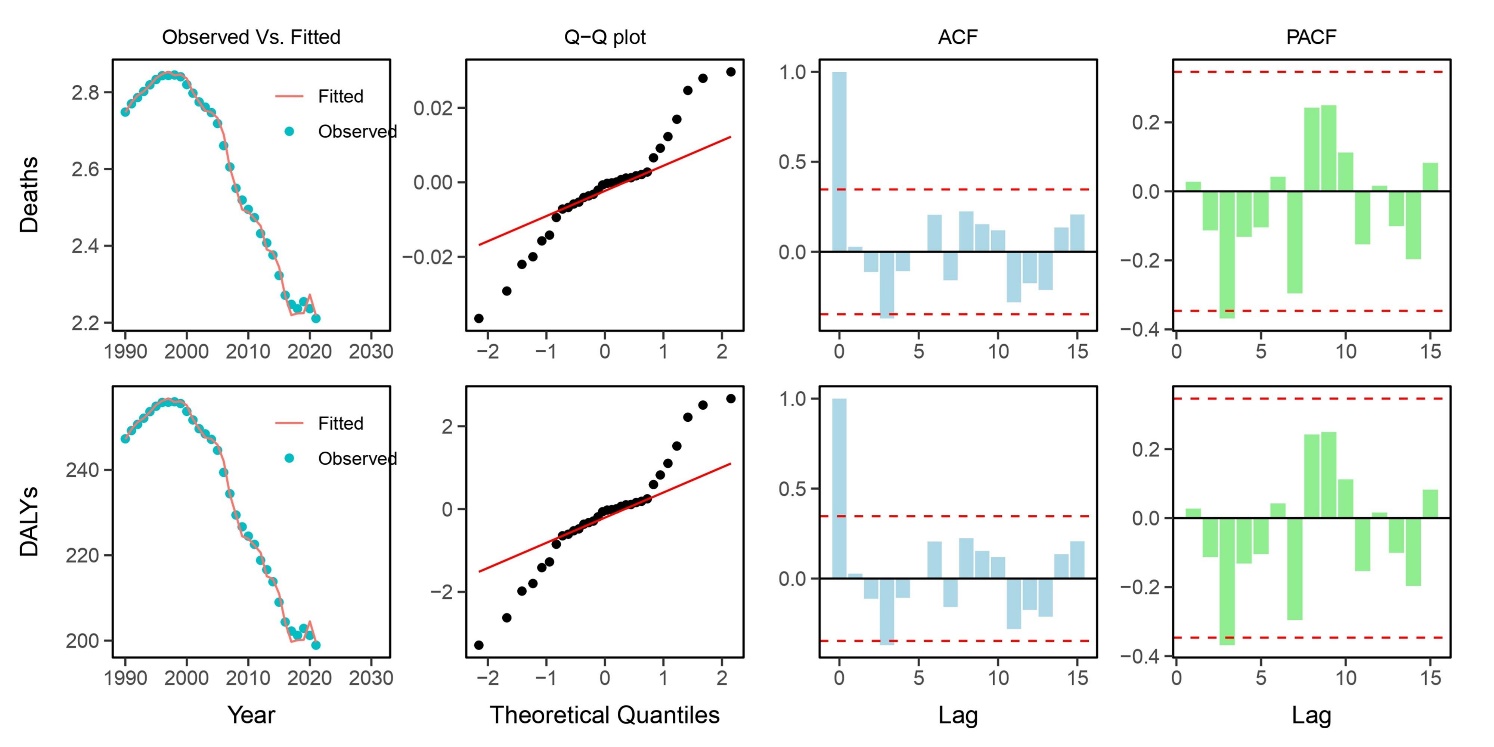
**

# Supplementary Figure S1.

Trend plot, *Q*-*Q* plot, Autocorrelation function (ACF) and Partial Autocorrelation Function plots (PACF) of Autoregressive Integrated Moving Average (ARIMA) Model in neonatal sepsis and other neonatal infections attributable to low birth weight.

# Supplementary Table S1.

The deaths burden of neonatal sepsis and other neonatal infections attributable to low birth weight in 204 countries and territories between 1990 and 2021.

| **Characteristics** | **Deaths cases** | | | **ASMR per 100,000 population** | | |
| --- | --- | --- | --- | --- | --- | --- |
|  | **1990 No. (95 %UI)** | **2021 No. (95 %UI)** | **EAPC No. (95 %CI)** | **1990 No. (95 %UI)** | **2021 No. (95 %UI)** | **EAPC No. (95 %CI)** |
| Afghanistan | 199.40 (120.77 to 308.86) | 492.11 (283.91 to 777.69) | -1.43 (-1.71 to -1.15) | 0.95 (0.58 to 1.48) | 0.85 (0.49 to 1.34) | -0.54 (-0.69 to -0.38) |
| Albania | 0.44 (0.25 to 0.76) | 0.31 (0.15 to 0.61) | -0.87 (-1.58 to -0.15) | 0.01 (0.01 to 0.02) | 0.02 (0.01 to 0.05) | 1.98 (0.99 to 2.98) |
| Algeria | 82.45 (35.20 to 162.28) | 48.74 (17.78 to 120.14) | -2.78 (-3.04 to -2.52) | 0.22 (0.10 to 0.44) | 0.11 (0.04 to 0.27) | -2.39 (-2.55 to -2.23) |
| American Samoa | 0.23 (0.15 to 0.33) | 0.03 (0.02 to 0.05) | -7.12 (-7.92 to -6.3) | 0.27 (0.18 to 0.39) | 0.10 (0.05 to 0.16) | -4.24 (-4.99 to -3.48) |
| Andorra | 0.03 (0.02 to 0.05) | 0.01 (0.00 to 0.01) | -5.11 (-5.49 to -4.73) | 0.11 (0.06 to 0.18) | 0.03 (0.01 to 0.05) | -3.01 (-3.38 to -2.64) |
| Angola | 869.09 (550.70 to 1,307.17) | 890.19 (591.71 to 1,335.71) | -3.67 (-3.97 to -3.37) | 3.64 (2.31 to 5.48) | 1.54 (1.02 to 2.31) | -2.91 (-3.08 to -2.75) |
| Antigua and Barbuda | 0.28 (0.22 to 0.36) | 0.28 (0.22 to 0.34) | -1.42 (-1.77 to -1.07) | 0.48 (0.37 to 0.61) | 0.55 (0.44 to 0.68) | 0.68 (0.38 to 0.97) |
| Argentina | 415.58 (348.11 to 485.68) | 131.62 (98.58 to 175.41) | -4.51 (-4.85 to -4.17) | 1.25 (1.04 to 1.46) | 0.51 (0.38 to 0.68) | -3.3 (-3.59 to -3) |
| Armenia | 87.57 (69.28 to 118.59) | 28.10 (22.77 to 34.51) | -3.06 (-3.36 to -2.75) | 2.41 (1.91 to 3.27) | 1.69 (1.37 to 2.07) | -2.03 (-2.55 to -1.5) |
| Australia | 29.45 (26.01 to 33.01) | 11.44 (9.32 to 13.94) | -4.23 (-5.1 to -3.35) | 0.24 (0.21 to 0.27) | 0.08 (0.07 to 0.10) | -3.65 (-4.54 to -2.76) |
| Austria | 3.41 (2.88 to 4.00) | 1.85 (1.48 to 2.24) | -1.88 (-2.44 to -1.31) | 0.08 (0.06 to 0.09) | 0.05 (0.04 to 0.05) | -1.18 (-1.58 to -0.78) |
| Azerbaijan | 57.99 (33.42 to 90.45) | 39.84 (24.00 to 68.25) | -1.63 (-2 to -1.26) | 0.65 (0.38 to 1.02) | 0.62 (0.37 to 1.06) | -0.33 (-0.75 to 0.08) |
| Bahamas | 3.39 (2.62 to 4.41) | 2.31 (1.70 to 3.09) | -2.61 (-3.04 to -2.17) | 1.28 (0.98 to 1.66) | 1.20 (0.88 to 1.61) | -0.41 (-0.75 to -0.06) |
| Bahrain | 1.87 (1.27 to 2.70) | 0.72 (0.50 to 1.04) | -6.00 (-6.22 to -5.78) | 0.29 (0.20 to 0.42) | 0.08 (0.06 to 0.12) | -3.67 (-3.86 to -3.48) |
| Bangladesh | 17,964.54 (12,033.28 to 25,408.27) | 5,226.89 (3,326.21 to 7,649.33) | -5.01 (-5.18 to -4.84) | 8.88 (5.94 to 12.53) | 3.89 (2.48 to 5.70) | -2.59 (-2.64 to -2.55) |
| Barbados | 2.44 (1.92 to 2.95) | 1.99 (1.39 to 2.82) | -0.67 (-1.28 to -0.05) | 1.22 (0.96 to 1.47) | 1.58 (1.10 to 2.23) | 1.38 (0.87 to 1.89) |
| Belarus | 34.08 (23.05 to 51.82) | 11.63 (8.51 to 15.27) | -1.47 (-2.06 to -0.87) | 0.50 (0.34 to 0.76) | 0.29 (0.21 to 0.38) | -1.48 (-1.75 to -1.21) |
| Belgium | 11.25 (9.78 to 12.89) | 6.67 (5.34 to 8.17) | -2.04 (-2.41 to -1.67) | 0.19 (0.16 to 0.21) | 0.12 (0.10 to 0.15) | -1.61 (-1.99 to -1.23) |
| Belize | 4.99 (4.05 to 6.07) | 5.06 (4.02 to 6.26) | -2.24 (-2.73 to -1.74) | 1.65 (1.34 to 2.01) | 1.37 (1.09 to 1.70) | -0.30 (-0.73 to 0.13) |
| Benin | 884.27 (618.18 to 1,228.58) | 1,206.23 (853.55 to 1,627.36) | -2.02 (-2.11 to -1.93) | 7.80 (5.46 to 10.82) | 4.82 (3.41 to 6.50) | -1.38 (-1.45 to -1.32) |
| Bermuda | 0.07 (0.05 to 0.09) | 0.02 (0.01 to 0.02) | -4.60 (-5.13 to -4.06) | 0.17 (0.13 to 0.21) | 0.07 (0.04 to 0.09) | -2.52 (-2.98 to -2.06) |
| Bhutan | 26.76 (17.66 to 38.21) | 4.97 (3.03 to 7.92) | -5.78 (-5.84 to -5.72) | 2.53 (1.67 to 3.60) | 0.83 (0.51 to 1.32) | -3.50 (-3.58 to -3.43) |
| Bolivia (Plurinational State of) | 345.34 (225.99 to 486.66) | 195.98 (134.55 to 275.84) | -3.53 (-3.67 to -3.38) | 3.17(2.07 to 4.47) | 1.67 (1.15 to 2.35) | -1.91 (-1.98 to -1.83) |
| Bosnia and Herzegovina | 3.01 (0.34 to 7.94) | 0.70 (0.15 to 1.52) | -3.72 (-4.08 to -3.37) | 0.09 (0.01 to 0.24) | 0.05 (0.01 to 0.11) | -2.22 (-2.77 to -1.67) |
| Botswana | 52.15 (35.00 to 77.72) | 50.82 (33.62 to 71.05) | -1.6 (-1.8 to -1.4) | 2.35 (1.58 to 3.50) | 2.18 (1.45 to 3.05) | 0.08 (-0.13 to 0.29) |
| Brazil | 4,454.16 (3,912.91 to 5,076.39) | 1,682.51 (1,321.07 to 2,140.18) | -4.09 (-4.53 to -3.64) | 2.88 (2.53 to 3.28) | 1.05 (0.83 to 1.34) | -2.95 (-3.39 to -2.5) |
| Brunei Darussalam | 2.31 (1.47 to 3.32) | 2.0 4(1.43 to 2.90) | -1.94 (-2.19 to -1.69) | 0.68 (0.44 to 0.98) | 0.69 (0.48 to 0.98) | 0.49 (0.31 to 0.67) |
| Bulgaria | 1.23 (0.98 to 1.54) | 1.88 (1.50 to 2.31) | 2.56 (0.64 to 4.51) | 0.03 (0.02 to 0.03) | 0.07 (0.05 to 0.08) | 2.69 (0.75 to 4.67) |
| Burkina Faso | 1,790.93 (1,162.53 to 2,523.87) | 2,584.14 (1,755.31 to 3,736.48) | -1.56 (-1.61 to -1.51) | 8.11 (5.26 to 11.41) | 5.69 (3.86 to 8.22) | -1.06 (-1.14 to -0.99) |
| Burundi | 794.49 (544.28 to 1,122.08) | 1,024.96 (672.70 to 1,533.05) | -1.53 (-1.78 to -1.28) | 6.40 (4.38 to 9.02) | 4.58 (3.00 to 6.84) | -0.91 (-1.04 to -0.78) |
| Cabo Verde | 14.23 (8.67 to 20.64) | 6.00 (3.75 to 9.21) | -4.04 (-4.22 to -3.86) | 2.36 (1.44 to 3.42) | 1.47 (0.92 to 2.26) | -1.45 (-1.58 to -1.32) |
| Cambodia | 768.43 (484.73 to 1,126.58) | 320.22 (205.44 to 467.07) | -4.69 (-4.82 to -4.57) | 3.76 (2.37 to 5.51) | 1.87 (1.20 to 2.73) | -2.78 (-3 to -2.56) |
| Cameroon | 1,250.60 (873.83 to 1,727.40) | 1,719.34 (1,190.03 to 2,351.47) | -2.24 (-2.44 to -2.04) | 5.49 (3.84 to 7.58) | 3.46 (2.40 to 4.73) | -1.43 (-1.5 to -1.36) |
| Canada | 18.61 (15.96 to 21.71) | 15.79 (12.53 to 19.81) | -1.22 (-1.45 to -0.99) | 0.10 (0.08 to 0.11) | 0.09 (0.07 to 0.11) | -0.23 (-0.49 to 0.02) |
| Central African Republic | 178.15 (110.96 to 284.22) | 269.48 (159.42 to 435.86) | -0.60 (-0.88 to -0.31) | 2.97 (1.85 to 4.72) | 2.98 (1.76 to 4.82) | 0.27 (0.07 to 0.47) |
| Chad | 1,121.06 (729.12 to 1,585.77) | 2,304.63 (1,546.98 to 3,500.11) | -1.12 (-1.16 to -1.08) | 7.36 (4.79 to 10.40) | 5.59 (3.75 to 8.48) | -0.84 (-0.86 to -0.81) |
| Chile | 59.97 (51.06 to 69.03) | 24.34 (19.51 to 29.67) | -3.01 (-3.65 to -2.37) | 0.41 (0.35 to 0.47) | 0.25 (0.20 to 0.30) | -1.00 (-1.57 to -0.42) |
| China | 2,998.83 (2,347.45 to 3,835.00) | 421.90 (311.70 to 528.49) | -5.75 (-6.3 to -5.19) | 0.27 (0.21 to 0.35) | 0.08 (0.06 to 0.10) | -4.06 (-4.33 to -3.8) |
| Colombia | 345.24 (295.35 to 403.54) | 331.59 (235.00 to 478.5) | -0.84 (-1.45 to -0.23) | 0.78 (0.67 to 0.91) | 1.02 (0.72 to 1.47) | 1.65 (0.97 to 2.33) |
| Comoros | 67.91 (44.09 to 97.92) | 39.54 (25.39 to 58.53) | -3.16 (-3.31 to -3.02) | 7.22 (4.70 to 10.42) | 4.86 (3.12 to 7.19) | -1.18 (-1.29 to -1.06) |
| Congo | 82.69 (43.36 to 137.23) | 108.83 (55.75 to 178.53) | -1.34 (-1.82 to -0.85) | 1.89 (0.99 to 3.14) | 1.78 (0.91 to 2.92) | -0.04 (-0.24 to 0.15) |
| Cook Islands | 0.08 (0.05 to 0.13) | 0.02 (0.01 to 0.04) | -6.20 (-6.94 to -5.45) | 0.40 (0.25 to 0.61) | 0.21 (0.07 to 0.39) | -4.06 (-4.83 to -3.28) |
| Costa Rica | 14.57 (11.38 to 17.75) | 7.58 (5.89 to 9.54) | -3.76 (-4.09 to -3.42) | 0.37 (0.29 to 0.45) | 0.29 (0.22 to0.36) | -1.59 (-2.04 to -1.15) |
| Côte d'Ivoire | 2,066.12 (1,468.99 to 2,837.02) | 2,078.32 (1,335.31 to 3,029.62) | -2.48 (-2.64 to -2.32) | 7.83 (5.57 to 10.73) | 4.59 (2.95 to 6.69) | -1.81 (-1.97 to -1.64) |
| Croatia | 1.84 (1.49 to 2.20) | 2.92 (2.05 to 4.02) | 0.79 (-0.96 to 2.57) | 0.07 (0.06 to 0.08) | 0.17 (0.12 to 0.24) | 1.64 (-0.15 to 3.46) |
| Cuba | 48.47 (40.55 to 59.28) | 12.77 (10.88 to 15.21) | -3.89 (-4.12 to -3.66) | 0.56 (0.47 to 0.69) | 0.26 (0.22 to 0.31) | -2.42 (-2.74 to -2.11) |
| Cyprus | 1.92 (1.18 to 3.13) | 0.62 (0.36 to 0.90) | -5.53 (-5.91 to -5.15) | 0.29 (0.18 to 0.47) | 0.08 (0.05 to 0.12) | -4.38 (-4.6 to -4.17) |
| Czechia | 2.93 (2.40 to 3.48) | 3.25 (2.55 to 4.15) | 1.25 (-0.12 to 2.64) | 0.05 (0.04 to 0.06) | 0.06 (0.05 to 0.08) | 1.06 (-0.35 to 2.49) |
| Democratic People's Republic of Korea | 66.11 (33.30 to 113.47) | 21.54 (9.57 to 40.72) | -4.37 (-4.52 to -4.23) | 0.26 (0.13 to 0.44) | 0.15 (0.07 to 0.29) | -1.53 (-1.69 to -1.38) |
| Democratic Republic of the Congo | 1,400.25 (820.78 to 2,188.11) | 2,090.33 (1,007.75 to 4,187.14) | -0.51 (-1.04 to 0.02) | 1.63 (0.96 to 2.55) | 1.53 (0.74 to 3.06) | 0.61 (0.20 to 1.02) |
| Denmark | 0.89 (0.67 to 1.12) | 0.68 (0.47 to 0.90) | -1.55 (-1.85 to -1.24) | 0.03 (0.02 to 0.04) | 0.02 (0.02 to 0.03) | -0.71 (-1.01 to -0.42) |
| Djibouti | 38.30 (26.04 to 56.64) | 47.19 (29.85 to 68.04) | -2.46 (-2.8 to -2.12) | 5.14 (3.50 to 7.59) | 3.26 (2.06 to 4.70) | -1.27 (-1.51 to -1.03) |
| Dominica | 1.61 (1.06 to 2.30) | 1.07 (0.71 to 1.55) | -0.51 (-0.86 to -0.16) | 1.79 (1.19 to 2.57) | 3.44 (2.28 to 5.01) | 2.66 (2.45 to 2.86) |
| Dominican Republic | 482.61 (360.09 to 628.52) | 449.67 (314.65 to 633.36) | -1.43 (-1.67 to -1.2) | 4.49 (3.35 to 5.85) | 4.39 (3.07 to 6.18) | 0.27 (-0.01 to 0.54) |
| Ecuador | 242.43 (197.13 to 298.49) | 116.59 (88.70 to 154.57) | -4.84 (-5.38 to -4.29) | 1.68 (1.36 to 2.06) | 0.75 (0.57 to 1.00) | -3.24 (-3.8 to -2.67) |
| Egypt | 262.13 (130.93 to 525.26) | 147.28 (86.99 to 230.80) | -2.79 (-3.32 to -2.25) | 0.29 (0.15 to 0.58) | 0.12 (0.07 to 0.19) | -2.69 (-2.99 to -2.38) |
| El Salvador | 162.94 (119.62 to 210.45) | 33.89 (22.48 to 49.79) | -5.68 (-5.86 to -5.49) | 1.95 (1.43 to 2.52) | 0.60 (0.40 to 0.89) | -3.73 (-3.87 to -3.6) |
| Equatorial Guinea | 24.68 (13.89 to 44.05) | 26.94 (15.20 to 41.39) | -4.1 (-4.25 to -3.95) | 2.52 (1.42 to 4.50) | 1.50 (0.85 to 2.30) | -2.10 (-2.24 to -1.96) |
| Eritrea | 383.85 (239.20 to 606.02) | 367.75 (231.97 to 549.86) | -2.08 (-2.25 to -1.9) | 5.43 (3.38 to 8.56) | 3.93 (2.48 to 5.88) | -0.95 (-1.15 to -0.75) |
| Estonia | 4.38 (3.71 to 5.14) | 1.06 (0.89 to 1.26) | -4.12 (-4.58 to -3.67) | 0.42 (0.36 to 0.50) | 0.17 (0.14 to 0.20) | -4.26 (-4.93 to -3.6) |
| Eswatini | 34.47 (22.11 to 52.83) | 24.71 (14.80 to 37.32) | -1.67 (-1.91 to -1.42) | 2.23 (1.43 to 3.42) | 1.78 (1.07 to 2.69) | -0.34 (-0.55 to -0.12) |
| Ethiopia | 11,165.75 (8,669.35 to 14,230.61) | 8,597.35 (6,000.85 to 11,484.02) | -3.33 (-3.52 to -3.14) | 9.66 (7.49 to 12.29) | 5.16 (3.60 to 6.89) | -1.90 (-2.05 to -1.76) |
| Fiji | 8.85 (6.12 to 12.42) | 5.74 (3.75 to 8.18) | -2.25 (-2.68 to -1.81) | 0.98 (0.68 to 1.37) | 0.66 (0.43 to 0.94) | -1.76 (-2.29 to -1.23) |
| Finland | 2.15 (1.71 to 2.68) | 1.02 (0.81 to 1.26) | -2.79 (-3.01 to -2.57) | 0.07 (0.05 to 0.08) | 0.04 (0.03 to 0.05) | -1.67 (-1.94 to -1.4) |
| France | 94.68 (83.93 to 106.70) | 53.44 (40.93 to 66.78) | -1.48 (-1.84 to -1.12) | 0.26 (0.23 to 0.29) | 0.16 (0.12 to 0.20) | -0.92 (-1.34 to -0.51) |
| Gabon | 30.00 (18.20 to 49.42) | 32.21 (19.32 to 48.66) | -1.05 (-1.41 to -0.68) | 1.76 (1.07 to 2.90) | 1.56 (0.94 to 2.36) | 0.17 (-0.06 to 0.4) |
| Gambia | 176.00 (124.43 to 244.10) | 167.99 (107.10 to 237.30) | -3.01 (-3.21 to -2.8) | 8.17 (5.78 to 11.31) | 4.54 (2.89 to 6.41) | -1.94 (-2.03 to -1.86) |
| Georgia | 15.58 (11.46 to 20.87) | 19.37 (14.91 to 25.34) | 3.8 (2.65 to 4.96) | 0.38 (0.28 to 0.50) | 0.90 (0.69 to 1.17) | 3.32 (2.16 to 4.5) |
| Germany | 45.36 (36.83 to 53.77) | 25.49 (21.03 to 30.42) | -1.88 (-2.35 to -1.40) | 0.11 (0.09 to 0.13) | 0.07 (0.06 to 0.08) | -1.53 (-1.81 to -1.24) |
| Ghana | 2,506.04 (1,793.20 to 3,456.73) | 2,383.27 (1,542.07 to 3,688.02) | -2.29 (-2.44 to -2.13) | 8.70 (6.23 to 11.99) | 5.13 (3.32 to 7.94) | -1.41 (-1.49 to -1.33) |
| Greece | 5.72 (4.38 to 7.06) | 2.06 (1.60 to 2.64) | -2.38 (-3.22 to -1.54) | 0.11 (0.09 to 0.14) | 0.05 (0.04 to 0.07) | -1.88 (-2.86 to -0.89) |
| Greenland | 0.02 (0.01 to 0.04) | 0.01 (0.01 to 0.02) | -1.89 (-2.95 to -0.81) | 0.04 (0.01 to 0.06) | 0.03 (0.01 to 0.05) | -0.54 (-1.38 to 0.3) |
| Grenada | 1.25 (0.90 to 1.65) | 0.74 (0.58 to 0.95) | -1.76 (-2.05 to -1.48) | 1.08 (0.78 to 1.42) | 1.15 (0.89 to 1.47) | 0.62 (0.34 to 0.9) |
| Guam | 0.10 (0.07 to 0.14) | 0.47 (0.31 to 0.69) | 5.33 (3.17 to 7.54) | 0.05 (0.04 to 0.08) | 0.37 (0.24 to 0.54) | 7.16 (4.8 to 9.57) |
| Guatemala | 412.89 (326.99 to 522.13) | 217.65 (165.82 to 286.52) | -4.21 (-4.47 to -3.95) | 2.49 (1.97 to 3.15) | 1.52 (1.16 to 2.00) | -1.71 (-1.98 to -1.44) |
| Guinea | 1,056.64 (724.06 to 1,499.90) | 1,122.62 (750.29 to 1,631.66) | -2.2 (-2.29 to -2.11) | 7.64 (5.25 to 10.82) | 4.72 (3.16 to 6.86) | -1.48 (-1.53 to -1.42) |
| Guinea-Bissau | 223.94 (151.98 to 308.28) | 179.18 (120.11 to 271.36) | -2.88 (-3.14 to -2.61) | 10.25 (6.96 to 14.08) | 5.22 (3.50 to 7.90) | -2.21 (-2.39 to -2.02) |
| Guyana | 30.79 (25.16 to 36.67) | 17.52 (12.18 to 24.30) | -2.2 (-2.71 to -1.67) | 2.47 (2.01 to 2.94) | 2.44 (1.70 to 3.39) | -0.44 (-1.1 to 0.23) |
| Haiti | 611.49 (352.21 to 952.13) | 800.03 (497.86 to 1,184.42) | -1.22 (-1.3 to -1.15) | 5.07 (2.92 to 7.89) | 4.89 (3.04 to 7.23) | -0.08 (-0.13 to -0.03) |
| Honduras | 244.82(163.82 to 354.58) | 202.14 (122.24 to 300.63) | -3.01 (-3.1 to -2.91) | 2.85 (1.91 to 4.13) | 1.89 (1.14 to 2.81) | -1.26 (-1.38 to -1.14) |
| Hungary | 2.86 (2.03 to 3.71) | 2.20 (1.57 to 2.90) | -0.54 (-1.63 to 0.56) | 0.05 (0.03 to 0.06) | 0.05 (0.04 to 0.07) | 0.06 (-1.17 to 1.3) |
| Iceland | 0.22 (0.18 to 0.27) | 0.07 (0.06 to 0.09) | -4.14 (-4.41 to -3.87) | 0.10 (0.08 to 0.12) | 0.03 (0.03 to 0.04) | -3.10 (-3.32 to -2.88) |
| India | 43,767.77 (36,420.68 to 52,915.03) | 23,821.34 (17,526.64 to 31,036.70) | -3.75 (-3.89 to -3.6) | 3.65 (3.04 to 4.42) | 2.27 (1.67 to 2.96) | -1.75 (-1.86 to -1.63) |
| Indonesia | 6,488.92 (4,235.03 to 9,707.92) | 3,170.85 (2,010.21 to 5,351.56) | -3.64 (-3.85 to -3.42) | 2.89 (1.89 to 4.32) | 1.50 (0.95 to 2.52) | -2.25 (-2.37 to -2.12) |
| Iran (Islamic Republic of) | 227.58 (170.57 to 288.34) | 50.07 (27.37 to 71.82) | -3.32 (-4.07 to -2.55) | 0.31 (0.23 to 0.39) | 0.10 (0.05 to 0.14) | -2.03 (-2.48 to -1.57) |
| Iraq | 771.56 (534.48 to 1,067.45) | 579.45 (353.78 to 834.34) | -3.78 (-4.00 to -3.55) | 2.33 (1.62 to 3.23) | 1.45 (0.89 to 2.09) | -2.04 (-2.24 to -1.84) |
| Ireland | 1.36 (1.08 to 1.64) | 1.17 (0.90 to 1.49) | -0.73 (-1.05 to -0.41) | 0.05 (0.04 to 0.06) | 0.04 (0.03 to 0.05) | -0.30 (-0.6 to 0) |
| Israel | 6.86 (5.56 to 8.65) | 5.47 (4.37 to 6.97) | -2.47 (-2.74 to -2.2) | 0.14 (0.11 to 0.17) | 0.06 (0.05 to 0.08) | -2.27 (-2.51 to -2.03) |
| Italy | 53.78 (49.64 to 57.44) | 31.01 (24.41 to 37.76) | -0.81 (-1.43 to -0.18) | 0.20 (0.18 to 0.21) | 0.16 (0.13 to 0.19) | 0.17 (-0.52 to 0.86) |
| Jamaica | 40.20 (31.46 to 50.63) | 29.70 (21.38 to 41.15) | -1.15 (-1.59 to -0.72) | 1.45 (1.14 to 1.83) | 1.87 (1.35 to 2.59) | 1.30 (0.91 to 1.69) |
| Japan | 65.61 (61.78 to 69.60) | 15.70 (13.32 to 18.24) | -4.96 (-5.47 to -4.44) | 0.11 (0.10 to 0.12) | 0.04 (0.03 to 0.04) | -3.86 (-4.32 to -3.39) |
| Jordan | 101.06 (75.08 to 134.70) | 84.30 (55.64 to 118.28) | -4.10 (-4.44 to -3.76) | 1.58 (1.18 to 2.11) | 0.81 (0.54 to 1.14) | -2.07 (-2.18 to -1.96) |
| Kazakhstan | 56.41 (36.69 to 79.79) | 45.28 (34.51 to 58.13) | -0.27 (-0.78 to 0.24) | 0.32 (0.21 to 0.45) | 0.23 (0.17 to 0.29) | -1.16 (-1.47 to -0.86) |
| Kenya | 2,032.50 (1,493.38 to 2,613.98) | 1,873.91 (1,413.03 to 2,447.49) | -2.15 (-2.52 to -1.78) | 4.31 (3.17 to 5.55) | 3.31 (2.49 to 4.32) | -0.40 (-0.61 to -0.19) |
| Kiribati | 0.96 (0.62 to 1.39) | 0.75 (0.47 to 1.08) | -2.33 (-2.45 to -2.21) | 0.74 (0.48 to 1.08) | 0.53 (0.33 to 0.77) | -1.19 (-1.39 to -0.99) |
| Kuwait | 4.87 (3.99 to 5.97) | 3.64 (2.76 to 4.70) | -4.00 (-4.88 to -3.11) | 0.30 (0.25 to 0.37) | 0.15 (0.11 to 0.20) | -2.04 (-3.28 to -0.79) |
| Kyrgyzstan | 11.16 (7.27 to 19.34) | 32.25 (26.45 to 39.32) | 2.99 (2.54 to 3.43) | 0.18 (0.11 to 0.31) | 0.43 (0.35 to 0.52) | 2.86 (2.41 to 3.3) |
| Lao People's Democratic Republic | 337.54 (199.05 to 504.99) | 180.61 (112.44 to 274.59) | -4.23 (-4.47 to -3.99) | 4.08 (2.41 to 6.11) | 2.14 (1.33 to 3.26) | -2.28 (-2.57 to -1.99) |
| Latvia | 5.30 (4.61 to 6.10) | 1.40 (1.18 to 1.67) | -2.13 (-2.48 to -1.78) | 0.30 (0.26 to 0.34) | 0.17 (0.14 to 0.21) | -2.15 (-2.5 to -1.79) |
| Lebanon | 17.15 (8.76 to 31.68) | 7.15 (4.28 to 11.08) | -4.45 (-4.6 to -4.29) | 0.42 (0.21 to 0.77) | 0.19 (0.11 to 0.29) | -2.54 (-2.75 to -2.33) |
| Lesotho | 82.38 (51.14 to 136.46) | 65.25 (45.03 to 93.85) | -1.13 (-1.28 to -0.98) | 3.22 (2.00 to 5.33) | 3.23 (2.23 to 4.64) | 0.16 (0.03 to 0.29) |
| Liberia | 437.93 (278.00 to 690.17) | 338.30 (212.15 to 511.25) | -3.34 (-3.62 to -3.06) | 8.18 (5.20 to 12.89) | 4.34 (2.72 to 6.56) | -2.20 (-2.38 to -2.02) |
| Libya | 13.99 (8.27 to 21.19) | 5.39 (2.25 to 13.88) | -4.56 (-4.70 to -4.42) | 0.22 (0.13 to 0.34) | 0.14 (0.06 to 0.36) | -1.61 (-1.9 to -1.32) |
| Lithuania | 6.53 (5.44 to 7.66) | 1.97 (1.63 to 2.42) | -2.22 (-2.78 to -1.64) | 0.24 (0.20 to 0.28) | 0.17 (0.14 to 0.21) | -1.32 (-1.58 to -1.06) |
| Luxembourg | 0.18 (0.16 to 0.22) | 0.10 (0.08 to 0.13) | -3.47 (-4.02 to -2.91) | 0.08 (0.06 to 0.09) | 0.03 (0.02 to 0.04) | -2.47 (-3.05 to -1.89) |
| Madagascar | 1,082.64 (794.77 to 1,450.67) | 1,479.99 (941.84 to 2,123.64) | -1.42 (-1.65 to -1.19) | 4.35 (3.19 to 5.82) | 3.66 (2.33 to 5.25) | -0.26 (-0.36 to -0.15) |
| Malawi | 1,443.49 (1,013.76 to 1,968.67) | 1,069.32 (723.04 to 1,525.01) | -3.18 (-3.48 to -2.88) | 6.05 (4.25 to 8.25) | 3.92 (2.65 to 5.59) | -1.42 (-1.5 to -1.34) |
| Malaysia | 296.50 (212.51 to 397.80) | 138.08 (96.76 to 183.95) | -4.24 (-4.72 to -3.75) | 1.24 (0.89 to 1.66) | 0.60 (0.42 to 0.80) | -2.16 (-2.51 to -1.81) |
| Maldives | 9.58 (6.06 to 13.92) | 2.15 (1.37 to 3.21) | -6.97 (-7.23 to -6.7) | 2.25 (1.42 to 3.27) | 0.74 (0.47 to 1.11) | -3.83 (-3.98 to -3.69) |
| Mali | 2,296.31 (1,527.39 to 3,283.56) | 2,495.71 (1,670.14 to 3,665.64) | -3.03 (-3.25 to -2.81) | 10.81 (7.21 to 15.46) | 4.90 (3.28 to 7.20) | -2.59 (-2.76 to -2.41) |
| Malta | 0.16 (0.14 to 0.20) | 0.09 (0.07 to 0.12) | -1.66 (-2.2 to -1.11) | 0.06 (0.05 to 0.07) | 0.04 (0.03 to 0.06) | -0.53 (-0.8 to -0.25) |
| Marshall Islands | 0.48 (0.32 to 0.73) | 0.30 (0.19 to 0.46) | -2.15 (-2.58 to -1.72) | 0.67 (0.44 to 1.01) | 0.55 (0.34 to 0.84) | -0.59 (-0.89 to -0.29) |
| Mauritania | 353.98 (246.60 to 498.57) | 272.50 (175.87 to 390.72) | -3.07 (-3.31 to -2.82) | 8.56 (5.97 to 12.05) | 4.20 (2.71 to 6.02) | -2.31 (-2.43 to -2.2) |
| Mauritius | 10.99 (9.36 to 12.93) | 5.62 (4.55 to 6.74) | -2.29 (-2.7 to -1.89) | 0.99 (0.84 to 1.16) | 0.92 (0.74 to 1.10) | 0.44 (-0.05 to 0.93) |
| Mexico | 1,870.30 (1,670.10 to 2,064.66) | 1,422.50 (1,142.61 to 1,759.81) | -2.3 (-2.51 to -2.1) | 1.54 (1.37 to 1.70) | 1.56 (1.26 to 1.94) | -0.28 (-0.45 to -0.1) |
| Micronesia (Federated States of) | 1.14 (0.71 to 1.78) | 0.39 (0.24 to 0.60) | -3.71 (-3.9 to -3.51) | 0.75 (0.47 to 1.17) | 0.43 (0.27 to 0.67) | -2.09 (-2.3 to -1.88) |
| Monaco | 0.02 (0.01 to 0.03) | 0.01 (0.01 to 0.02) | -2.11 (-2.37 to -1.84) | 0.12 (0.08 to 0.20) | 0.08 (0.05 to 0.12) | -1.93 (-2.2 to -1.67) |
| Mongolia | 15.99 (8.67 to 28.01) | 15.54 (8.83 to 24.02) | -0.70 (-1.08 to -0.33) | 0.46 (0.25 to 0.81) | 0.42 (0.24 to 0.65) | -0.51 (-0.63 to -0.39) |
| Montenegro | 1.35 (0.82 to 2.01) | 0.35 (0.18 to 0.63) | -4.62 (-5.01 to -4.22) | 0.28 (0.17 to 0.42) | 0.10 (0.05 to 0.18) | -3.64 (-4.11 to -3.17) |
| Morocco | 166.53 (95.98 to 260.58) | 73.61 (43.08 to 116.93) | -3.50 (-3.84 to -3.16) | 0.44 (0.25 to 0.69) | 0.24 (0.14 to 0.38) | -1.89 (-2.10 to -1.68) |
| Mozambique | 2,827.74 (1,936.08 to 3,772.90) | 2,873.50 (1,676.67 to 4,444.61) | -2.67 (-2.82 to -2.52) | 9.73 (6.66 to 12.97) | 5.43 (3.17 to 8.39) | -1.83 (-1.91 to -1.76) |
| Myanmar | 3,219.72 (2,149.91 to 4,482.28) | 1,735.96 (1,149.89 to 2,613.23) | -3.10 (-3.17 to -3.02) | 6.03 (4.03 to 8.39) | 3.38 (2.24 to 5.08) | -2.04 (-2.17 to -1.92) |
| Namibia | 67.71 (46.41 to 101.73) | 56.03 (34.96 to 84.45) | -1.75 (-1.99 to -1.51) | 2.76 (1.89 to 4.14) | 2.03 (1.27 to 3.07) | -0.55 (-0.71 to -0.38) |
| Nauru | 0.09 (0.06 to 0.13) | 0.06 (0.06 to 0.10) | -1.68 (-2.27 to -1.09) | 0.53 (0.33 to 0.79) | 0.45 (0.26 to 0.72) | -0.83 (-1.37 to -0.29) |
| Nepal | 1,436.80 (921.79 to 2,159.61) | 598.73 (390.49 to 914.37) | -4.45 (-4.53 to -4.36) | 3.84 (2.47 to 5.76) | 1.94 (1.27 to 2.97) | -2.11 (-2.19 to -2.04) |
| Netherlands | 23.35 (20.43 to 26.47) | 18.64 (15.68 to 21.36) | -1.01 (-1.27 to -0.76) | 0.25 (0.21 to 0.28) | 0.22 (0.18 to 0.25) | 0.02 (-0.24 to 0.28) |
| New Zealand | 2.15 (1.82 to 2.50) | 2.66 (2.21 to 3.17) | 1.59 (0.24 to 2.96) | 0.07 (0.06 to 0.09) | 0.09 (0.07 to 0.11) | 2.78 (1.46 to 4.12) |
| Nicaragua | 243.98 (195.25 to 309.63) | 101.80 (71.45 to 140.63) | -4.20 (-4.32 to -4.08) | 3.57 (2.86 to 4.54) | 1.65 (1.16 to 2.28) | -2.43 (-2.58 to -2.28) |
| Niger | 1,860.58 (1,247.57 to 2,700.93) | 3,058.69 (1,829.58 to 4,708.86) | -2.53 (-2.76 to -2.3) | 8.76 (5.88 to 12.69) | 5.40 (3.24 to 8.32) | -1.88 (-2.05 to -1.71) |
| Nigeria | 12,655.62 (9,831.23 to 15,734.41) | 20,102.69 (15,115.29 to 26,122.90) | -1.37 (-1.6 to -1.14) | 6.51 (5.06 to 8.09) | 5.10 (3.83 to 6.62) | -0.66 (-0.76 to -0.56) |
| Niue | 0.01 (0.01 to 0.01) | 0.01 (0.01 to 0.02) | -0.81 (-1.78 to 0.16) | 0.43 (0.28 to 0.62) | 1.12 (0.75 to 1.61) | 0.20 (-0.79 to 1.2) |
| North Macedonia | 0.32(0.01 to 0.64) | 0.30 (0.12 to 0.58) | 0.72 (-0.03 to 1.47) | 0.02 (0.01 to 0.04) | 0.03 (0.01 to 0.06) | 2.72 (1.83 to 3.61) |
| Northern Mariana Islands | 0.08 (0.05 to 0.12) | 0.02 (0.01 to 0.02) | -5.27 (-6.31 to -4.22) | 0.13 (0.08 to 0.20) | 0.06 (0.04 to 0.08) | -2.20 (-3.16 to -1.23) |
| Norway | 2.24 (2.02 to 2.48) | 0.56 (0.46 to 0.68) | -3.86 (-5.83 to -1.84) | 0.08 (0.07 to 0.09) | 0.02 (0.02 to 0.03) | -2.87 (-4.81 to -0.9) |
| Oman | 13.50 (8.24 to 20.71) | 7.23 (4.38 to 10.93) | -3.77 (-4.42 to -3.12) | 0.39 (0.24 to 0.60) | 0.19 (0.11 to 0.29) | -1.95 (-2.35 to -1.55) |
| Pakistan | 6,706.12 (4,820.13 to 9,090.56) | 8,770.76 (6,167.88 to 11,858.23) | -1.63 (-1.76 to -1.49) | 3.35 (2.41 to 4.54) | 2.95 (2.07 to 3.98) | -0.63 (-0.77 to -0.49) |
| Palau | 0.03 (0.02 to 0.04) | 0.01 (0.01 to 0.02) | -3.52 (-3.83 to -3.22) | 0.18 (0.11 to 0.29) | 0.13 (0.07 to 0.22) | -1.37 (-1.62 to -1.11) |
| Palestine | 31.76 (20.88 to 46.47) | 26.35 (17.14 to 38.12) | -3.25 (-3.5 to -3) | 0.75 (0.49 to 1.10) | 0.45 (0.30 to 0.66) | -1.35 (-1.5 to -1.2) |
| Panama | 44.16 (36.61 to 52.57) | 37.02 (28.52 to 47.57) | -2.07 (-2.39 to -1.76) | 1.55 (1.29 to 1.85) | 1.09 (0.84 to 1.40) | -1.00 (-1.23 to -0.76) |
| Papua New Guinea | 70.69 (39.51 to 111.73) | 163.95 (90.88 to 269.86) | -0.32 (-0.54 to -0.1) | 1.00 (0.56 to 1.58) | 1.00 (0.56 to 1.65) | -0.02 (-0.26 to 0.22) |
| Paraguay | 192.98 (148.33 to 245.38) | 48.95 (31.79 to 69.29) | -6.84 (-7.18 to -6.5) | 3.11 (2.39 to 3.95) | 0.79 (0.51 to 1.12) | -5.21 (-5.6 to -4.82) |
| Peru | 1,360.86 (964.94 to 1,836.85) | 589.55 (386.20 to 846.33) | -3.09 (-3.48 to -2.7) | 4.41 (3.13 to 5.96) | 1.82 (1.19 to 2.61) | -1.65 (-2.02 to -1.27) |
| Philippines | 3,922.73 (3,206.84 to 4,733.48) | 2,929.82 (2,208.86 to 3,730.63) | -2.45 (-2.78 to -2.11) | 4.05 (3.31 to 4.88) | 2.68 (2.02 to 3.42) | -1.07 (-1.33 to -0.81) |
| Poland | 94.16 (75.97 to 115.01) | 8.16 (6.40 to 10.33) | -8.65 (-9.40 to -7.90) | 0.36 (0.29 to 0.44) | 0.05 (0.04 to 0.06) | -7.92 (-8.86 to -6.97) |
| Portugal | 19.62 (16.41 to 22.64) | 5.31 (4.37 to 6.38) | -3.50 (-3.85 to -3.15) | 0.35 (0.30 to 0.41) | 0.13 (0.11 to 0.16) | -2.21 (-2.62 to -1.81) |
| Puerto Rico | 19.97 (17.44 to 22.67) | 5.81 (4.76 to 7.18) | -3.28 (-4.53 to -2.02) | 0.63 (0.55 to 0.72) | 0.65 (0.54 to 0.81) | 0.41 (-0.58 to 1.41) |
| Qatar | 0.45 (0.30 to 0.67) | 0.49 (0.29 to 0.72) | -6.09 (-6.39 to -5.78) | 0.08 (0.05 to 0.12) | 0.03 (0.02 to 0.04) | -3.67 (-3.83 to -3.51) |
| Republic of Korea | 121.89 (91.14 to 161.27) | 14.15 (9.23 to 19.48) | -6.92 (-7.17 to -6.67) | 0.38 (0.28 to 0.50) | 0.11 (0.07 to 0.15) | -3.68 (-4.07 to -3.29) |
| Republic of Moldova | 60.74 (48.72 to 72.34) | 20.72 (15.03 to 27.83) | -2.16 (-2.81 to -1.50) | 1.59 (1.28 to 1.90) | 1.52 (1.10 to 2.04) | -0.43 (-0.94 to 0.08) |
| Romania | 11.81 (8.18 to 15.02) | 2.30 (1.85 to 2.82) | -4.69 (-5.43 to -3.93) | 0.08 (0.06 to 0.10) | 0.03 (0.02 to 0.03) | -4.24 (-5.09 to -3.38) |
| Russian Federation | 406.32 (379.44 to 431.23) | 303.39 (267.53 to 343.10) | 0.10 (-0.47 to 0.68) | 0.43 (0.40 to 0.45) | 0.46 (0.41 to 0.52) | -0.47 (-1.1 to 0.16) |
| Rwanda | 1,057.13 (769.86 to 1,461.75) | 544.56 (352.92 to 777.73) | -3.96 (-4.26 to -3.66) | 6.97 (5.08 to 9.62) | 3.08 (1.99 to 4.39) | -2.38 (-2.58 to -2.18) |
| Saint Kitts and Nevis | 0.18 (0.14 to 0.22) | 0.14 (0.10 to 0.18) | -1.80 (-2.04 to -1.56) | 0.40 (0.32 to 0.48) | 0.49 (0.37 to 0.64) | 0.99 (0.65 to 1.33) |
| Saint Lucia | 2.03 (1.64 to 2.48) | 1.16 (0.82 to 1.60) | -2.23 (-2.65 to -1.8) | 1.19 (0.96 to 1.44) | 1.43 (1.01 to 1.97) | 1.13 (0.59 to 1.67) |
| Saint Vincent and the Grenadines | 1.96 (1.51 to 2.48) | 0.94 (0.68 to 1.24) | -2.05 (-2.57 to -1.52) | 1.58 (1.22 to 2.01) | 1.50 (1.09 to 1.98) | 0.07 (-0.43 to 0.58) |
| Samoa | 0.82 (0.45 to 1.35) | 0.63 (0.33 to 0.98) | -1.51 (-1.62 to -1.39) | 0.32 (0.17 to 0.52) | 0.21 (0.11 to 0.33) | -1.22 (-1.37 to -1.07) |
| San Marino | 0.03 (0.02 to 0.05) | 0.01 (0.00 to 0.02) | -4.25 (-4.54 to -3.95) | 0.28 (0.16 to 0.45) | 0.09 (0.03 to 0.18) | -2.93 (-3.22 to -2.64) |
| Sao Tome and Principe | 9.29 (6.10 to 13.96) | 4.67 (2.80 to 7.80) | -4.03 (-4.52 to -3.54) | 4.20 (2.76 to 6.30) | 1.97 (1.18 to 3.29) | -2.44 (-2.65 to -2.22) |
| Saudi Arabia | 247.59 (139.22 to 398.17) | 47.68 (26.14 to 74.59) | -8.06 (-8.44 to -7.67) | 1.01 (0.57 to 1.62) | 0.21 (0.12 to 0.33) | -5.36 (-5.76 to -4.95) |
| Senegal | 1,228.45 (855.94 to 1,698.74) | 982.82 (688.62 to 1,359.66) | -2.64 (-2.8 to -2.47) | 7.45 (5.19 to 10.30) | 4.26 (2.99 to 5.90) | -1.54 (-1.65 to -1.44) |
| Serbia | 14.15 (7.70 to 24.31) | 2.26 (1.51 to 3.10) | -6.76 (-7.41 to -6.1) | 0.21 (0.12 to 0.37) | 0.07 (0.05 to 0.10) | -4.73 (-5.37 to -4.08) |
| Seychelles | 1.17 (0.80 to 1.69) | 0.87 (0.54 to 1.29) | -1.65 (-1.89 to -1.41) | 1.48 (1.01 to 2.14) | 1.14 (0.70 to 1.69) | -0.57 (-0.7 to -0.45) |
| Sierra Leone | 966.20 (609.69 to 1,398.52) | 846.59 (560.65 to 1,222.72) | -2.65 (-2.99 to -2.31) | 10.00 (6.32 to 14.45) | 5.86 (3.88 to 8.46) | -1.59 (-1.85 to -1.34) |
| Singapore | 1.09 (0.93 to 1.26) | 0.68 (0.53 to 0.85) | -3.54 (-3.98 to -3.1) | 0.04 (0.04 to 0.05) | 0.03 (0.02 to 0.03) | -1.78 (-2.14 to -1.43) |
| Slovakia | 0.95 (0.64 to 1.35) | 0.54 (0.37 to 0.74) | -1.53 (-1.98 to -1.09) | 0.02 (0.02 to 0.04) | 0.02 (0.01 to 0.03) | -0.90 (-1.56 to -0.24) |
| Slovenia | 0.08 (0.04 to 0.11) | 0.09 (0.05 to 0.12) | 1.23 (-0.08 to 2.57) | 0.01 (0.00 to 0.01) | 0.01 (0.01 to 0.01) | 1.23 (-0.12 to 2.59) |
| Solomon Islands | 6.05 (3.54 to 9.87) | 6.52 (3.87 to 11.05) | -2.06 (-2.32 to -1.79) | 0.92 (0.54 to 1.51) | 0.67 (0.40 to 1.13) | -1.03 (-1.23 to -0.83) |
| Somalia | 1,265.61 (689.34 to 1,885.10) | 2,759.21 (1,375.20 to 4,468.03) | -0.57 (-0.77 to -0.37) | 6.87 (3.74 to 10.23) | 6.01 (2.99 to 9.72) | -0.34 (-0.45 to -0.23) |
| South Africa | 1,054.73 (803.22 to 1,326.22) | 999.29 (772.00 to 1,235.46) | -1.15 (-1.42 to -0.89) | 2.10 (1.60 to 2.64) | 2.11 (1.63 to 2.61) | 0.05 (-0.11 to 0.22) |
| South Sudan | 762.69 (459.51 to 1,138.36) | 952.97 (479.36 to 1,713.23) | -0.79 (-1.02 to -0.56) | 6.25 (3.76 to 9.33) | 5.22 (2.63 to 9.38) | -0.36 (-0.49 to -0.24) |
| Spain | 51.69 (46.03 to 58.12) | 27.74 (23.40 to 32.53) | -1.59 (-2.24 to -0.93) | 0.27 (0.24 to 0.30) | 0.17 (0.14 to 0.20) | -0.96 (-1.33 to -0.59) |
| Sri Lanka | 270.00 (207.06 to 350.48) | 85.07 (59.62 to 119.09) | -4.37 (-4.74 to -3.99) | 1.56 (1.20 to 2.03) | 0.58 (0.41 to 0.82) | -3.30 (-3.58 to -3.02) |
| Sudan | 254.75 (153.88 to 401.54) | 203.43 (119.47 to 320.04) | -3.08 (-3.29 to -2.87) | 0.61 (0.37 to 0.97) | 0.37 (0.22 to 0.58) | -1.61 (-1.66 to -1.55) |
| Suriname | 13.11 (9.38 to 18.53) | 10.52 (6.76 to 15.14) | -1.68 (-1.85 to -1.5) | 2.99 (2.14 to 4.22) | 2.46 (1.58 to 3.54) | -0.42 (-0.59 to -0.24) |
| Sweden | 7.67 (6.64 to 8.85) | 3.23 (2.68 to 3.85) | -2.69 (-3.37 to -2) | 0.13 (0.11 to 0.15) | 0.06 (0.05 to 0.07) | -2.47 (-3.15 to -1.77) |
| Switzerland | 5.57 (4.78 to 6.42) | 5.12 (4.04 to 6.34) | -1.20 (-1.47 to -0.92) | 0.14 (0.12 to 0.16) | 0.12 (0.10 to 0.15) | -0.62 (-0.89 to -0.35) |
| Syrian Arab Republic | 132.41 (76.78 to 223.63) | 18.65 (9.01 to 36.00) | -5.97 (-6.31 to -5.63) | 0.60 (0.35 to 1.02) | 0.20 (0.10 to 0.38) | -3.02 (-3.29 to -2.75) |
| Taiwan (Province of China) | 1.69 (1.43 to 1.98) | 12.92 (10.73 to 15.65) | 7.50 (6.63 to 8.39) | 0.01 (0.01 to 0.01) | 0.17 (0.14 to 0.21) | 10.55 (9.57 to 11.53) |
| Tajikistan | 53.55 (27.93 to 91.44) | 61.14 (32.16 to 107.35) | -1.28 (-1.4 to -1.15) | 0.54 (0.28 to 0.91) | 0.45 (0.24 to 0.79) | -0.63 (-0.84 to -0.42) |
| Thailand | 893.77 (543.32 to 1,412.89) | 142.72 (108.52 to 181.58) | -6.01 (-6.27 to -5.74) | 1.79 (1.09 to 2.83) | 0.55 (0.42 to 0.70) | -3.53 (-3.76 to -3.29) |
| Timor-Leste | 70.96 (45.40 to 100.57) | 45.93 (31.21 to 65.64) | -3.80 (-4.04 to -3.57) | 4.27 (2.73 to 6.04) | 2.34 (1.59 to 3.35) | -2.24 (-2.4 to -2.08) |
| Togo | 516.24 (357.85 to 741.81) | 441.09 (288.95 to 647.95) | -2.82 (-3.03 to -2.60) | 6.74 (4.68 to 9.68) | 3.79 (2.48 to 5.57) | -1.74 (-1.81 to -1.67) |
| Tokelau | 0.01 (0.01 to 0.02) | 0.03 (0.01 to 0.05) | -1.68 (-3.27 to -0.06) | 0.71 (0.34 to 1.26) | 2.96 (1.00 to 5.28) | 0.04 (-1.61 to 1.71) |
| Tonga | 0.71 (0.37 to 1.18) | 0.43 (0.21 to 0.75) | -1.62 (-1.86 to -1.37) | 0.46 (0.24 to 0.75) | 0.30 (0.15 to 0.52) | -1.23 (-1.48 to -0.99) |
| Trinidad and Tobago | 23.85 (18.94 to 29.07) | 13.94 (10.33 to 18.93) | -1.54 (-1.82 to -1.25) | 2.08 (1.65 to 2.53) | 1.95 (1.44 to 2.65) | -0.30 (-0.56 to -0.05) |
| Tunisia | 27.44 (15.16 to 45.00) | 10.81 (5.60 to 21.22) | -3.27 (-3.52 to -3.03) | 0.26 (0.14 to 0.43) | 0.13 (0.07 to 0.26) | -1.92 (-2.02 to -1.83) |
| Turkey | 1,264.32 (859.79 to 1,768.25) | 288.82 (179.57 to 422.46) | -5.56 (-5.7 to -5.41) | 1.78 (1.21 to 2.50) | 0.59 (0.37 to 0.87) | -3.55 (-3.69 to -3.42) |
| Turkmenistan | 24.46 (17.33 to 33.80) | 21.98 (15.61 to 30.25) | -0.72 (-1.01 to -0.43) | 0.41 (0.29 to 0.56) | 0.41 (0.29 to 0.57) | 0.59 (0.31 to 0.87) |
| Tuvalu | 0.08 (0.04 to 0.15) | 0.03 (0.02 to 0.06) | -3.42 (-3.5 to -3.33) | 0.48 (0.24 to 0.89) | 0.27 (0.15 to 0.48) | -1.84 (-1.98 to -1.7) |
| Uganda | 2,404.87 (1,730.99 to 3,296.93) | 2,958.14 (2,047.11 to 4,150.68) | -2.16 (-2.39 to -1.93) | 5.47 (3.94 to 7.49) | 3.91 (2.71 to 5.49) | -0.95 (-1.09 to -0.81) |
| Ukraine | 112.31 (81.53 to 150.12) | 44.79 (34.09 to 56.59) | -0.69 (-1.23 to -0.15) | 0.35 (0.25 to 0.46) | 0.34 (0.26 to 0.43) | 0.15 (-0.06 to 0.36) |
| United Arab Emirates | 1.43 (0.60 to 3.33) | 1.29 (0.73 to 2.65) | -3.77 (-4.34 to -3.2) | 0.06 (0.03 to 0.14) | 0.03 (0.02 to 0. 07) | -0.57 (-0.97 to -0.17) |
| United Kingdom | 33.05 (28.33 to 37.23) | 10.70 (8.64 to12.77) | -4.34 (-4.75 to -3.93) | 0.09 (0.07 to 0.10) | 0.03 (0.03 to 0.04) | -3.80 (-4.26 to -3.35) |
| United Republic of Tanzania | 3,379.13 (2,326.15 to 4,715.74) | 4,368.97 (2,847.50 to 6,736.94) | -1.28 (-1.49 to -1.08) | 5.88 (4.05 to 8.20) | 4.84 (3.15 to 7.46) | -0.16 (-0.35 to 0.03) |
| United States of America | 374.15 (347.24 to 402.02) | 305.32 (270.38 to 339.72) | -1.33 (-1.73 to -0.94) | 0.19 (0.17 to 0.20) | 0.17 (0.15 to 0.19) | -0.24 (-0.57 to 0.09) |
| United States Virgin Islands | 0.50 (0.35 to 0.71) | 0.11 (0.06 to 0.17) | -3.71 (-3.84 to -3.59) | 0.45 (0.31 to 0.63) | 0.30 (0.17 to 0.48) | -0.66 (-0.81 to -0.51) |
| Uruguay | 30.64 (26.31 to 34.66) | 8.95 (7.03 to 11.65) | -4.44 (-4.78 to -4.09) | 1.14 (0.98 to 1.29) | 0.52 (0.41 to 0.68) | -2.95 (-3.31 to -2.58) |
| Uzbekistan | 115.36 (83.67 to 174.46) | 266.38 (212.83 to 323.97) | 1.97 (1.48 to 2.46) | 0.34(0.25 to 0.51) | 0.69 (0.56 to 0.85) | 2.79 (2.30 to 3.27) |
| Vanuatu | 1.71 (0.95 to 2.74) | 1.94 (1.13 to 3.14) | -2.01 (-2.39 to -1.62) | 0.59 (0.33 to 0.94) | 0.46 (0.27 to 0.75) | -0.89 (-1.23 to -0.54) |
| Venezuela (Bolivarian Republic of) | 522.94 (445.63 to 611.23) | 315.34 (216.86 to 455.55) | -1.96 (-2.31 to -1.61) | 1.98 (1.69 to 2.31) | 1.45 (1.00 to 2.10) | -0.71 (-1.02 to -0.4) |
| Viet Nam | 2,383.30 (1,592.60 to 3,427.99) | 654.23 (425.23 to 995.37) | -5.13 (-5.35 to -4.92) | 2.57 (1.72 to 3.69) | 0.88 (0.57 to 1.33) | -3.78 (-3.9 to -3.65) |
| Yemen | 177.24 (108.69 to 276.48) | 195.84 (114.16 to 297.13) | -2.27 (-2.56 to -1.98) | 0.57 (0.35 to 0.88) | 0.42 (0.25 to 0.64) | -0.84 (-0.91 to -0.78) |
| Zambia | 905.31 (638.03 to 1,231.61) | 1,112.64 (723.49 to 1,634.05) | -1.92 (-2.32 to -1.52) | 4.96 (3.50 to 6.75) | 3.84 (2.50 to 5.64) | -0.66 (-0.87 to -0.45) |
| Zimbabwe | 478.07 (331.52 to 679.18) | 658.31 (472.33 to 944.63) | 0.76 (0.33 to 1.19) | 2.58 (1.79 to 3.66) | 2.94 (2.11 to 4.22) | 1.17 (0.87 to 1.47) |

ASMR, age-standardized mortality rate; EAPC, estimated annual percentage change; UI, uncertainty interval; CI, confidence interval.

Supplementary Table S2.

The deaths burden of neonatal sepsis and other neonatal infections attributable to low birth weight in 204 countries and territories between 1990 and 2021.

| **Characteristics** | **DALYs cases** | | | **ASDR per 100,000 population** | | |
| --- | --- | --- | --- | --- | --- | --- |
|  | **1990 No. (95 %UI)** | **2021 No. (95 %UI)** | **EAPC No. (95 %CI)** | **1990 No. (95 %UI)** | **2021 No. (95 %UI)** | **EAPC No. (95 %CI)** |
| Afghanistan | 17,941.11 (10,866.18 to 27,789.26) | 44,277.17 (25,544.73 to 69,971.97) | -1.43 (-1.71 to -1.15) | 85.61 (51.85 to 132.86) | 76.28 (44.01 to 120.54) | -0.54 (-0.69 to -0.38) |
| Albania | 39.26 (22.73 to 67.95) | 28.23 (13.65 to 54.57) | -0.87 (-1.58 to -0.15) | 1.01 (0.58 to 1.75) | 2.11 (1.02 to 4.08) | 1.98 (0.99 to 2.98) |
| Algeria | 7,418.41 (3,166.64 to 14,601.32) | 4,384.92 (1,599.73 to 10,809.90) | -2.78 (-3.04 to -2.52) | 20.09 (8.58 to 39.57) | 10.02 (3.65 to 24.70) | -2.39 (-2.55 to -2.23) |
| American Samoa | 20.47 (13.50 to 29.26) | 2.84 (1.57 to 4.67) | -7.12 (-7.92 to -6.3) | 24.25 (15.99 to 34.67) | 8.79 (4.85 to 14.44) | -4.24 (-4.99 to -3.48) |
| Andorra | 2.56 (1.43 to 4.05) | 0.55(0.18 to 1.01) | -5.11 (-5.49 to -4.73) | 10.05 (5.62 to 15.93) | 2.45 (0.80 to 4.55) | -3.01 (-3.38 to -2.64) |
| Angola | 78,195.24(49,549.00 to 117,611.46) | 80,094.71 (53,238.83 to 120,179.70) | -3.67 (-3.97 to -3.37) | 327.83 (207.79 to 493.08) | 138.29 (91.95 to 207.50) | -2.91 (-3.08 to -2.75) |
| Antigua and Barbuda | 25.47 (19.56 to 32.21) | 24.88 (19.77 to 30.77) | -1.42 (-1.77 to -1.07) | 43.41 (33.34 to 54.91) | 49.44 (39.27 to 61.12) | 0.68 (0.38 to 0.97) |
| Argentina | 37,391.22 (31,321.09 to 43,698.59) | 11,842.16 (8,869.94 to 15,781.99) | -4.51 (-4.85 to -4.17) | 112.02 (93.83 to 130.92) | 45.82 (34.32 to 61.06) | -3.3 (-3.59 to -3) |
| Armenia | 7,879.21 (6,233.23 to 10,670.42) | 2,528.27 (2,048.44 to 3,104.94) | -3.06 (-3.36 to -2.75) | 216.94 (171.62 to 293.80) | 151.87 (123.02 to 186.50) | -2.03 (-2.55 to -1.5) |
| Australia | 2,649.93 (2,339.89 to 2,970.23) | 1,029.30 (838.27 to 1,254.31) | -4.23 (-5.1 to -3.35) | 21.40 (18.89 to 23.98) | 7.20 (5.86 to 8.77) | -3.65 (-4.54 to -2.76) |
| Austria | 306.74 (258.85 to 360.27) | 166.65 (133.40 to 201.61) | -1.88 (-2.44 to -1.31) | 6.85 (5.78 to 8.05) | 4.06 (3.25 to 4.91) | -1.18 (-1.58 to -0.78) |
| Azerbaijan | 5,217.74 (3,007.25 to 8,138.00) | 3,584.20 (2,159.51 to 6,140.54) | -1.63 (-2 to -1.26) | 58.76 (33.86 to 91.67) | 55.59 (33.49 to 95.25) | -0.33 (-0.75 to 0.08) |
| Bahamas | 305.12 (235.39 to 396.95) | 207.53 (152.99 to 278.45) | -2.61 (-3.04 to -2.17) | 114.81 (88.57 to 149.37) | 107.70 (79.41 to 144.48) | -0.41 (-0.75 to -0.06) |
| Bahrain | 168.46 (114.65 to 242.85) | 64.77 (45.11 to 93.65) | -6.00 (-6.22 to -5.78) | 26.34 (17.92 to 37.97) | 7.61 (5.30 to 11.01) | -3.67 (-3.86 to -3.48) |
| Bangladesh | 1,616,353.53 (1,082,691.25 to 2,286,106.24) | 470,288.70 (299,274.81 to 688,248.96) | -5.01 (-5.18 to -4.84) | 798.84 (534.84 to 1,127.61) | 350.38 (222.96 to 512.78) | -2.59 (-2.64 to -2.55) |
| Barbados | 219.30 (172.78 to 265.50) | 179.32 (124.85 to 253.77) | -0.67 (-1.28 to -0.05) | 109.42 (86.20 to 132.47) | 141.74 (98.68 to 200.60) | 1.38 (0.87 to 1.89) |
| Belarus | 3,066.61 (2,073.92 to 4,662.82) | 1,046.65 (766.06 to 1,374.34) | -1.47 (-2.06 to -0.87) | 44.69 (30.21 to 67.94) | 26.09 (19.09 to 34.27) | -1.48 (-1.75 to -1.21) |
| Belgium | 1,012.51 (880.05 to 1,159.42) | 600.09 (480.13 to 734.97) | -2.04 (-2.41 to -1.67) | 16.76 (14.56 to 19.19) | 10.85 (8.68 to 13.29) | -1.61 (-1.99 to -1.23) |
| Belize | 449.20 (364.35 to 546.08) | 455.02 (361.32 to 563.41) | -2.24 (-2.73 to -1.74) | 148.47 (120.43 to 180.49) | 123.37 (97.94 to 152.74) | -0.30 (-0.73 to 0.13) |
| Benin | 79,561.43 (55,620.11 to 110,541.25) | 108,530.65 (76,798.15 to 146,421.24) | -2.02 (-2.11 to -1.93) | 701.36 (491.12 to 973.86) | 433.47 (306.65 to 584.84) | -1.38 (-1.45 to -1.32) |
| Bermuda | 6.47 (4.81 to 8.26) | 1.52 (0.93 to 2.01) | -4.60 (-5.13 to -4.06) | 15.14 (11.26 to 19.34) | 6.45 (3.94 to 8.54) | -2.52 (-2.98 to -2.06) |
| Bhutan | 2,408.01 (1,588.52 to 3,438.00) | 447.56 (272.36 to 712.27) | -5.78 (-5.84 to -5.72) | 227.34 (149.94 to 323.77) | 74.77 (45.50 to 119.00) | -3.50 (-3.58 to -3.43) |
| Bolivia (Plurinational State of) | 31,071.50 (20,333.11 to 43,787.37) | 17,633.31 (12,106.21 to 24,818.65) | -3.53 (-3.67 to -3.38) | 284.92 (186.49 to 401.78) | 150.46 (103.28 to 211.85) | -1.91 (-1.98 to -1.83) |
| Bosnia and Herzegovina | 270.73 (30.42 to 714.69) | 63.17 (13.63 to 136.55) | -3.72 (-4.08 to -3.37) | 8.32 (0.93 to 21.97) | 4.70 (1.02 to 10.17) | -2.22 (-2.77 to -1.67) |
| Botswana | 4,692.51 (3,149.47 to 6,992.65) | 4,572.32 (3,025.36 to 6,392.62) | -1.6 (-1.8 to -1.4) | 211.37 (141.92 to 314.87) | 196.50 (130.02 to 274.72) | 0.08 (-0.13 to 0.29) |
| Brazil | 400,759.53 (352,060.72 to 456,744.49) | 151,382.85 (118,862.69 to 192,561.45) | -4.09 (-4.53 to -3.64) | 258.84 (227.39 to 295.00) | 94.86 (74.49 to 120.66) | -2.95 (-3.39 to -2.5) |
| Brunei Darussalam | 207.74 (132.66 to 298.63) | 183.88 (129.08 to 261.31) | -1.94 (-2.19 to -1.69) | 61.34 (39.16 to 88.20) | 62.10 (43.57 to 88.29) | 0.49 (0.31 to 0.67) |
| Bulgaria | 110.61 (88.24 to 138.51) | 169.58 (134.59 to 207.50) | 2.56 (0.64 to 4.51) | 2.30 (1.84 to 2.88) | 6.05 (4.80 to 7.40) | 2.69 (0.75 to 4.67) |
| Burkina Faso | 161,138.15 (104,598.13 to 227,085.18) | 232,507.23 (157,933.57 to 336,188.93) | -1.56 (-1.61 to -1.51) | 729.73 (473.44 to 1,026.49) | 511.61 (347.64 to 739.56) | -1.06 (-1.14 to -0.99) |
| Burundi | 71,484.14 (48,971.66 to 100,958.68) | 92,220.55 (60,525.50 to 137,936.26) | -1.53 (-1.78 to -1.28) | 575.62 (394.31 to 811.99) | 411.85 (270.37 to 615.83) | -0.91 (-1.04 to -0.78) |
| Cabo Verde | 1,280.24 (779.88 to 1,856.76) | 539.57 (337.08 to 828.93) | -4.04 (-4.22 to -3.86) | 212.43 (129.41 to 308.10) | 132.25 (82.58 to 203.16) | -1.45 (-1.58 to -1.32) |
| Cambodia | 69,138.85 (43,613.32 to 101,363.41) | 28,812.06 (18,484.14 to 42,024.90) | -4.69 (-4.82 to -4.57) | 337.89 (213.26 to 495.85) | 168.26 (107.94 to 245.42) | -2.78 (-3 to -2.56) |
| Cameroon | 112,522.27 (78,621.98 to 155,422.09) | 154,697.11 (107,072.72 to 211,573.23) | -2.24 (-2.44 to -2.04) | 493.98 (345.57 to 681.71) | 311.34 (215.53 to 425.75) | -1.43 (-1.5 to -1.36) |
| Canada | 1,674.13 (1,435.95 to 1,953.55) | 1,421.12 (1,127.03 to 1,782.26) | -1.22 (-1.45 to -0.99) | 8.56 (7.34 to 9.99) | 7.98 (6.33 to 10.01) | -0.23 (-0.49 to 0.02) |
| Central African Republic | 16,029.17 (9,983.30 to 25,573.00) | 24,246.57 (14,343.52 to 39,215.98) | -0.60 (-0.88 to -0.31) | 266.85 (166.34 to 425.06) | 267.95 (158.51 to 433.73) | 0.27 (0.07 to 0.47) |
| Chad | 100,866.75 (65,602.26 to 142,679.40) | 207,358.32 (139,188.91 to 314,921.42) | -1.12 (-1.16 to -1.08) | 662.38 (430.69 to 935.42) | 502.77 (337.72 to 762.82) | -0.84 (-0.86 to -0.81) |
| Chile | 5,395.65 (4,594.11 to 6,211.21) | 2,190.13 (1,755.01 to 2,669.95) | -3.01 (-3.65 to -2.37) | 36.74 (31.27 to 42.30) | 22.39 (17.94 to 27.29) | -1.00 (-1.57 to -0.42) |
| China | 269,818.50 (211,210.61 to 345,053.06) | 37,960.454 (28,045.43 to 47,550.37) | -5.75 (-6.3 to -5.19) | 24.38 (19.08 to 31.18) | 7.12 (5.26 to 8.92) | -4.06 (-4.33 to -3.8) |
| Colombia | 31,062.94 (26,573.63 to 36,308.59) | 29,834.26 (21,143.97 to 43,059.80) | -0.84 (-1.45 to -0.23) | 70.26 (60.10 to 82.12) | 91.88 (65.13 to 132.65) | 1.65 (0.97 to 2.33) |
| Comoros | 6,109.79 (3,966.87 to 8,809.98) | 3,557.30 (2,284.52 to 5,266.40) | -3.16 (-3.31 to -3.02) | 649.90 (422.57 to 937.38) | 436.90 (280.71 to 646.56) | -1.18 (-1.29 to -1.06) |
| Congo | 7,440.01 (3,901.33 to 12,347.67) | 9,791.75 (5,016.51 to 16,062.81) | -1.34 (-1.82 to -0.85) | 170.37 (89.38 to 282.66) | 160.27 (82.11 to 262.96) | -0.04 (-0.24 to 0.15) |
| Cook Islands | 7.60 (4.85 to 11.55) | 1.99 (0.69 to 3.70) | -6.20 (-6.94 to -5.45) | 35.86 (22.87 to 54.48) | 18.75 (6.55 to 34.90) | -4.06 (-4.83 to -3.28) |
| Costa Rica | 1,311.01 (1,023.53 to 1,597.36) | 681.98 (530.23 to 858.02) | -3.76 (-4.09 to -3.42) | 33.44 (26.11 to 40.75) | 25.89 (20.13 to 32.58) | -1.59 (-2.04 to -1.15) |
| Côte d'Ivoire | 185,897.88 (132,171.11 to 255,260.58) | 186,996.47 (120,143.81 to 272,590.10) | -2.48 (-2.64 to -2.32) | 704.42 (501.01 to 965.24) | 412.89 (265.41 to 601.54) | -1.81 (-1.97 to -1.64) |
| Croatia | 165.49 (134.09 to 197.96) | 262.76 (184.78 to 361.88) | 0.79 (-0.96 to 2.57) | 6.18 (5.00 to 7.39) | 15.48 (10.89 to 21.32) | 1.64 (-0.15 to 3.46) |
| Cuba | 4,361.20 (3,648.83 to 5,333.26) | 1,148.99 (978.80 to 1,368.81) | -3.89 (-4.12 to -3.66) | 50.79 (42.49 to 62.12) | 23.74 (20.23 to 28.28) | -2.42 (-2.74 to -2.11) |
| Cyprus | 173.12 (106.22 to 281.75) | 55.37 (32.76 to 81.23) | -5.53 (-5.91 to -5.15) | 26.17 (16.05 to 42.60) | 7.59 (4.49 to 11.14) | -4.38 (-4.6 to -4.17) |
| Czechia | 263.64 (215.86 to 313.45) | 292.79 (229.53 to 373.50) | 1.25 (-0.12 to 2.64) | 4.25 (3.48 to 5.05) | 5.69 (4.46 to 7.26) | 1.06 (-0.35 to 2.49) |
| Democratic People's Republic of Korea | 5,947.82 (2,996.25 to 10,209.80) | 1,937.86 (861.41 to 3,663.97) | -4.37 (-4.52 to -4.23) | 23.04 (11.60 to 39.55) | 13.67 (6.08 to 25.84) | -1.53 (-1.69 to -1.38) |
| Democratic Republic of the Congo | 125,987.43 (73,849.18 to 196,875.00) | 188,076.71 (90,671.67 to 376,737.06) | -0.51 (-1.04 to 0.02) | 147.03 (86.23 to 229.59) | 137.66 (66.36 to 275.73) | 0.61 (0.20 to 1.02) |
| Denmark | 79.74 (60.20 to 100.79) | 60.83 (42.53 to 81.22) | -1.55 (-1.85 to -1.24) | 2.60 (1.96 to 3.28) | 1.98 (1.38 to 2.64) | -0.71 (-1.01 to -0.42) |
| Djibouti | 3,445.87 (2,342.56 to 5,096.16) | 4,246.25 (2,686.06 to 6,121.62) | -2.46 (-2.8 to -2.12) | 462.27 (314.63 to 683.28) | 293.23 (185.49 to 422.72) | -1.27 (-1.51 to -1.03) |
| Dominica | 144.48 (95.63 to 207.21) | 96.01 (63.58 to 139.82) | -0.51 (-0.86 to -0.16) | 161.15 (106.62 to 231.16) | 309.32 (204.84 to 450.45) | 2.66 (2.45 to 2.86) |
| Dominican Republic | 43,422.65 (32,399.17 to 56,551.10) | 40,458.74 (28,310.53 to 56,986.81) | -1.43 (-1.67 to -1.2) | 403.86 (301.53 to 526.03) | 394.59 (276.11 to 555.84) | 0.27 (-0.01 to 0.54) |
| Ecuador | 21,812.12 (17,736.31 to 26,856.32) | 10,490.11 (7,981.09 to 13,907.69) | -4.84 (-5.38 to -4.29) | 150.83 (122.65 to 185.70) | 67.93 (51.68 to 90.06) | -3.24 (-3.8 to -2.67) |
| Egypt | 23,584.97 (11,780.70 to 47,260.30) | 13,251.78 (7,826.57 to 20,766.10) | -2.79 (-3.32 to -2.25) | 26.19 (13.08 to 52.48) | 10.65 (6.30 to 16.69) | -2.69 (-2.99 to -2.38) |
| El Salvador | 14,660.30 (10,762.68 to 18,934.84) | 3,049.06 (2,023.02 to 4,479.92) | -5.68 (-5.86 to -5.49) | 175.47 (128.82 to 226.63) | 54.34 (36.05 to 79.82) | -3.73 (-3.87 to -3.6) |
| Equatorial Guinea | 2,220.52 (1,249.30 to 3,963.06) | 2,423.88 (1,368.03 to 3,723.91) | -4.1 (-4.25 to -3.95) | 226.75 (127.66 to 404.49) | 134.84 (76.11 to 207.17) | -2.10 (-2.24 to -1.96) |
| Eritrea | 34,536.80 (21,522.35 to 54,526.02) | 33,087.76 (20,871.07 to 49,473.66) | -2.08 (-2.25 to -1.9) | 488.22 (304.08 to 770.40) | 353.55 (223.03 to 528.63) | -0.95 (-1.15 to -0.75) |
| Estonia | 394.18 (333.71 to 462.75) | 95.46 (80.25 to 112.97) | -4.12 (-4.58 to -3.67) | 37.98 (32.14 to 44.59) | 15.23 (12.80 to 18.03) | -4.26 (-4.93 to -3.6) |
| Eswatini | 3,101.47 (1,988.96 to 4,753.77) | 2,223.41 (1,331.21 to 3,357.63) | -1.67 (-1.91 to -1.42) | 200.60 (128.65 to 307.41) | 160.20 (95.93 to 241.94) | -0.34 (-0.55 to -0.12) |
| Ethiopia | 1,004,631.24 (780,019.77 to 1,280,392.78) | 773,543.22 (539,924.25 to 1,033,269.87) | -3.33 (-3.52 to -3.14) | 868.87 (674.25 to 1,105.81) | 464.07 (323.91 to 619.90) | -1.90 (-2.05 to -1.76) |
| Fiji | 796.31 (550.66 to 1,117.41) | 516.05 (337.01 to 736.41) | -2.25 (-2.68 to -1.81) | 87.94 (60.81 to 123.39) | 59.35 (38.74 to 84.71) | -1.76 (-2.29 to -1.23) |
| Finland | 193.12 (154.11 to 240.81) | 91.39 (72.64 to 113.00) | -2.79 (-3.01 to -2.57) | 6.10 (4.87 to 7.61) | 3.92 (3.11 to 4.85) | -1.67 (-1.94 to -1.4) |
| France | 8,518.71 (7,551.35 to 9,600.44) | 4,808.08 (3,682.73 to 6,008.76) | -1.48 (-1.84 to -1.12) | 23.07 (20.45 to 26.00) | 14.43 (11.05 to 18.04) | -0.92 (-1.34 to -0.51) |
| Gabon | 2,699.46 (1,637.34 to 4,446.56) | 2,898.49 (1,738.43 to 4,377.79) | -1.05 (-1.41 to -0.68) | 158.41 (96.12 to 260.79) | 140.31 (84.15 to 211.93) | 0.17 (-0.06 to 0.4) |
| Gambia | 15,835.73 (11,195.24 to 21,962.51) | 15,115.28 (9,636.44 to 21,350.84) | -3.01 (-3.21 to -2.8) | 734.83 (520.20 to 1,017.88) | 408.21 (260.30 to 576.54) | -1.94 (-2.03 to -1.86) |
| Georgia | 1,402.10 (1,031.53 to 1,878.17) | 1,742.92 (1,341.17 to 2,280.28) | 3.8 (2.65 to 4.96) | 33.80 (24.87 to 45.28) | 80.78 (62.16 to 105.67) | 3.32 (2.16 to 4.5) |
| Germany | 4,081.38 (3,313.41 to 4,838.08) | 2,293.19 (1,892.21 to 2,736.64) | -1.88 (-2.35 to -1.40) | 9.60 (7.80 to 11.39) | 6.04 (4.99 to 7.22) | -1.53 (-1.81 to -1.24) |
| Ghana | 225,479.65 (161,342.35 to 311,018.49) | 214,434.09 (138,747.13 to 331,829.05) | -2.29 (-2.44 to -2.13) | 782.43 (560.46 to 1,078.42) | 461.88 (298.91 to 714.66) | -1.41 (-1.49 to -1.33) |
| Greece | 514.36 (394.00 to 635.46) | 185.46 (143.68 to 237.56) | -2.38 (-3.22 to -1.54) | 10.18 (7.80 to 12.58) | 4.64 (3.60 to 5.95) | -1.88 (-2.86 to -0.89) |
| Greenland | 1.92 (0.77 to 3.36) | 0.96 (0.49 to 1.58) | -1.89 (-2.95 to -0.81) | 3.32 (1.33 to 5.80) | 2.55 (1.31 to 4.22) | -0.54 (-1.38 to 0.3) |
| Grenada | 112.55 (81.24 to 148.18) | 66.98 (51.81 to 85.76) | -1.76 (-2.05 to -1.48) | 96.99 (70.01 to 127.72) | 103.12 (79.76 to 132.05) | 0.62 (0.34 to 0.9) |
| Guam | 8.86 (6.03 to 12.49) | 42.29 (27.76 to 61.81) | 5.33 (3.17 to 7.54) | 4.86 (3.31 to 6.85) | 33.26 (21.84 to 48.64) | 7.16 (4.8 to 9.57) |
| Guatemala | 37,149.50 (29,421.08 to 46,978.61) | 19,583.13 (14,919.17 to 25,779.12) | -4.21 (-4.47 to -3.95) | 224.10 (177.51 to 283.42) | 137.00 (104.37 to 180.33) | -1.71 (-1.98 to -1.44) |
| Guinea | 95,071.12 (65,146.76 to 134,953.30) | 101,007.12 (67,506.64 to 146,808.34) | -2.2 (-2.29 to -2.11) | 687.18 (472.15 to 973.77) | 424.80 (284.08 to 617.25) | -1.48 (-1.53 to -1.42) |
| Guinea-Bissau | 20,148.50 (13,674.24 to 27,737.09) | 16,121.91 (10,806.42 to 24,415.70) | -2.88 (-3.14 to -2.61) | 922.13 (626.52 to 1,266.93) | 469.69 (314.91 to 711.00) | -2.21 (-2.39 to -2.02) |
| Guyana | 2,770.62 (2,263.38 to 3,299.36) | 1,576.03 (1,095.91 to 2,186.00) | -2.2 (-2.71 to -1.67) | 221.79 (181.18 to 264.08) | 219.68 (152.76 to 304.69) | -0.44 (-1.1 to 0.23) |
| Haiti | 55,018.36 (31,689.55 to 85,667.43) | 71,982.78 (44,794.51 to 106,568.16) | -1.22 (-1.3 to -1.15) | 456.46 (263.06 to 710.32) | 439.75 (273.65 to 650.94) | -0.08 (-0.13 to -0.03) |
| Honduras | 22,027.39 (14,739.52 to 31,903.28) | 18,187.41 (10,998.54 to 27,049.45) | -3.01 (-3.1 to -2.91) | 256.54 (171.68 to 371.54) | 170.05 (102.82 to 253.02) | -1.26 (-1.38 to -1.14) |
| Hungary | 257.20 (182.70 to 334.02) | 198.38 (141.33 to 260.72) | -0.54 (-1.63 to 0.56) | 4.25 (3.02 to 5.52) | 4.62 (3.29 to 6.08) | 0.06 (-1.17 to 1.3) |
| Iceland | 20.14 (16.29 to 24.09) | 6.65 (5.21 to 8.35) | -4.14 (-4.41 to -3.87) | 9.02 (7.29 to 10.79) | 2.98 (2.33 to 3.74) | -3.10 (-3.32 to -2.88) |
| India | 3,937,991.23 (3,276,934.24 to 4,761,009.91) | 2,143,319.40 (1,576,954.54 to 2,792,520.49) | -3.75 (-3.89 to -3.6) | 328.82 (273.86 to 397.68) | 204.27 (150.29 to 266.14) | -1.75 (-1.86 to -1.63) |
| Indonesia | 583,837.29 (381,044.73 to 873,464.67) | 285,295.94 (180,868.00 to 481,505.34) | -3.64 (-3.85 to -3.42) | 260.03 (169.66 to 389.08) | 134.55 (85.29 to 227.12) | -2.25 (-2.37 to -2.12) |
| Iran (Islamic Republic of) | 20,476.02 (15,346.77 to 25,942.81) | 4,505.30 (2,462.61 to 6,462.00) | -3.32 (-4.07 to -2.55) | 27.61 (20.69 to 34.97) | 9.03 (4.94 to 12.96) | -2.03 (-2.48 to -1.57) |
| Iraq | 69,420.81 (48,089.76 to 96,043.13) | 52,135.80 (31,830.71 to 75,069.05) | -3.78 (-4.00 to -3.55) | 209.76 (145.32 to 290.19) | 130.66 (79.77 to 188.12) | -2.04 (-2.24 to -1.84) |
| Ireland | 122.30 (96.96 to 147.86) | 105.36 (81.40 to 134.31) | -0.73 (-1.05 to -0.41) | 4.66 (3.69 to 5.63) | 3.87 (2.99 to 4.94) | -0.30 (-0.6 to 0) |
| Israel | 617.58 (500.26 to 778.05) | 491.90 (392.96 to 627.13) | -2.47 (-2.74 to -2.2) | 12.18 (9.87 to 15.35) | 5.52 (4.41 to 7.04) | -2.27 (-2.51 to -2.03) |
| Italy | 4,838.62 (4,466.23 to 5,167.70) | 2,790.38 (2,195.94 to 3,397.24) | -0.81 (-1.43 to -0.18) | 17.92 (16.54 to 19.14) | 14.31 (11.26 to 17.42) | 0.17 (-0.52 to 0.86) |
| Jamaica | 3,617.10 (2,830.81 to 4,554.98) | 2,672.14 (1,923.57 to 3,702.76) | -1.15 (-1.59 to -0.72) | 130.70 (102.29 to 164.59) | 168.31 (121.14 to 233.22) | 1.30 (0.91 to 1.69) |
| Japan | 5,903.36 (5,558.78 to 6,262.48) | 1,412.92 (1,198.70 to 1,641.08) | -4.96 (-5.47 to -4.44) | 9.82 (9.24 to 10.41) | 3.40 (2.89 to 3.95) | -3.86 (-4.32 to -3.39) |
| Jordan | 9,092.46 (6,755.27 to 12,119.91) | 7,584.59 (5,006.32 to 10,641.76) | -4.10 (-4.44 to -3.76) | 142.41 (105.82 to 189.85) | 73.03 (48.21 to 102.48) | -2.07 (-2.18 to -1.96) |
| Kazakhstan | 5,075.43 (3,300.93 to 7,179.02) | 4,073.76 (3,104.86 to 5,230.60) | -0.27 (-0.78 to 0.24) | 28.53 (18.55 to 40.34) | 20.64 (15.73 to 26.49) | -1.16 (-1.47 to -0.86) |
| Kenya | 182,874.85 (134,366.04 to 235,191.88) | 168,604.97 (127,137.45 to 220,212.77) | -2.15 (-2.52 to -1.78) | 388.11 (285.22 to 499.11) | 297.56 (224.38to 388.64) | -0.40 (-0.61 to -0.19) |
| Kiribati | 86.82 (55.45 to 125.42) | 67.57 (41.87 to 97.40) | -2.33 (-2.45 to -2.21) | 66.97 (42.78 to 96.74) | 48.05 (29.77 to 69.26) | -1.19 (-1.39 to -0.99) |
| Kuwait | 437.89 (359.31 to 536.81) | 327.76 (248.26 to 423.02) | -4.00 (-4.88 to -3.11) | 27.06 (22.20 to 33.17) | 13.62 (10.32 to 17.58) | -2.04 (-3.28 to -0.79) |
| Kyrgyzstan | 1,004.39 (654.40 to 1,739.89) | 2,902.08 (2,380.19 to 3,537.46) | 2.99 (2.54 to 3.43) | 15.85 (10.32 to 27.46) | 38.50 (31.58 to 46.93) | 2.86 (2.41 to 3.3) |
| Lao People's Democratic Republic | 30,370.14 (17,909.69 to 45,436.47) | 16,250.19 (10,116.35 to 24,705.89) | -4.23 (-4.47 to -3.99) | 367.09 (216.78 to 549.41) | 192.94 (120.12 to 293.32) | -2.28 (-2.57 to -1.99) |
| Latvia | 477.19 (414.80 to 548.77) | 126.04 (106.16 to 150.32) | -2.13 (-2.48 to -1.78) | 26.88 (23.37 to 30.91) | 15.49 (13.05 to 18.48) | -2.15 (-2.5 to -1.79) |
| Lebanon | 1,543.44 (788.23 to 2,850.74) | 643.15 (385.16 to 996.89) | -4.45 (-4.6 to -4.29) | 37.73 (19.27 to 69.68) | 16.88 (10.10 to 26.18) | -2.54 (-2.75 to -2.33) |
| Lesotho | 7,411.72 (4,601.20 to 12,277.60) | 5,871.02 (4,051.53 to 8,443.91) | -1.13 (-1.28 to -0.98) | 289.84 (180.05 to 479.99) | 290.43 (200.49 to 417.56) | 0.16 (0.03 to 0.29) |
| Liberia | 39,402.68 (25,013.24 to 62,097.47) | 30,438.29 (19,087.62 to 45,999.45) | -3.34 (-3.62 to -3.06) | 735.99 (467.50 to 1,160.00) | 390.62 (245.09 to 590.19) | -2.20 (-2.38 to -2.02) |
| Libya | 1,259.13 (744.02 to 1,906.42) | 484.83 (202.07 to 1,248.55) | -4.56 (-4.70 to -4.42) | 20.14 (11.90 to 30.49) | 12.56 (5.23 to 32.34) | -1.61 (-1.9 to -1.32) |
| Lithuania | 587.26 (489.44 to 689.44) | 177.64 (146.98 to 217.89) | -2.22 (-2.78 to -1.64) | 21.59 (18.00 to 25.35) | 15.44 (12.78 to 18.94) | -1.32 (-1.58 to -1.06) |
| Luxembourg | 16.50 (13.95 to 19.67) | 9.38 (7.24 to 11.86) | -3.47 (-4.02 to -2.91) | 6.79 (5.74 to 8.10) | 2.87 (2.22 to 3.63) | -2.47 (-3.05 to -1.89) |
| Madagascar | 97,409.76 (71,508.60 to 130,523.56) | 133,160.96 (84,741.57 to 191,074.27) | -1.42 (-1.65 to -1.19) | 390.95 (287.40 to 523.72) | 328.94 (209.33 to 471.98) | -0.26 (-0.36 to -0.15) |
| Malawi | 129,877.75 (91,213.11 to 177,130.24) | 96,211.52 (65,055.69 to 137,212.39) | -3.18 (-3.48 to -2.88) | 544.39 (382.32 to 742.24) | 352.82 (238.59 to 503.14) | -1.42 (-1.5 to -1.34) |
| Malaysia | 26,677.55 (19,120.19 to 35,792.21) | 12,423.82 (8,706.30 to 16,550.77) | -4.24 (-4.72 to -3.75) | 111.44 (79.87 to 149.56) | 53.94 (37.78 to 71.89) | -2.16 (-2.51 to -1.81) |
| Maldives | 862.33 (544.86 to 1,252.64) | 193.47 (123.16 to 289.26) | -6.97 (-7.23 to -6.7) | 202.88 (128.19 to 294.56) | 66.80 (42.50 to 99.90) | -3.83 (-3.98 to -3.69) |
| Mali | 206,609.98 (137,425.85 to 295,437.54) | 224,551.58 (150,270.20 to 329,815.98) | -3.03 (-3.25 to -2.81) | 973.01 (648.80 to 1,390.58 | 441.18 (295.41 to 647.60) | -2.59 (-2.76 to -2.41) |
| Malta | 14.83 (12.27 to 17.64) | 8.36 (6.51 to 10.64) | -1.66 (-2.2 to -1.11) | 5.53 (4.58 to 6.58) | 3.98 (3.10 to 5.07) | -0.53 (-0.8 to -0.25) |
| Marshall Islands | 43.06 (28.64 to 65.63) | 27.39 (16.83 to 41.63) | -2.15 (-2.58 to -1.72) | 59.89 (39.83 to 91.32) | 49.88 (30.65 to 75.82) | -0.59 (-0.89 to -0.29) |
| Mauritania | 31,849.44 (22,187.66 to 44,859.16) | 24,519.46 (15,823.37 to 35,155.22) | -3.07 (-3.31 to -2.82) | 770.56 (537.48 to 1,083.98) | 377.93 (243.90 to 541.87) | -2.31 (-2.43 to -2.2) |
| Mauritius | 988.50 (841.85 to 1,163.14) | 505.69 (409.31 to 606.53) | -2.29 (-2.7 to -1.89) | 88.96 (75.76 to 104.68) | 82.68 (66.92 to 99.15) | 0.44 (-0.05 to 0.93) |
| Mexico | 168,279.04 (150,266.60 to 185,766.35) | 127,988.36 (102,805.37 to 158,337.84) | -2.3 (-2.51 to -2.1) | 138.20 (123.41 to 152.56) | 140.79 (113.10 to 174.20) | -0.28 (-0.45 to -0.1) |
| Micronesia (Federated States of) | 102.68 (64.30 to 159.76) | 34.86 (21.61 to 53.77) | -3.71 (-3.9 to -3.51) | 67.80 (42.44 to 105.51) | 38.88 (24.11 to 59.98) | -2.09 (-2.3 to -1.88) |
| Monaco | 1.39 (0.88 to 2.34) | 1.13 (0.75 to 1.64) | -2.11 (-2.37 to -1.84) | 10.70 (6.76 to 18.04) | 7.34 (4.87 to 10.60) | -1.93 (-2.2 to -1.67) |
| Mongolia | 1,438.50 (780.37 to 2,520.49) | 1,398.10 (794.24 to 2,161.14) | -0.70 (-1.08 to -0.33) | 41.55 (22.54 to 72.79) | 38.02 (21.59 to 58.77) | -0.51 (-0.63 to -0.39) |
| Montenegro | 121.13 (74.02 to 180.81) | 31.94 (16.21 to 56.49) | -4.62 (-5.01 to -4.22) | 25.27 (15.44 to 37.72) | 9.41 (4.77 to 16.65) | -3.64 (-4.11 to -3.17) |
| Morocco | 14,983.12 (8,635.85 to 23,445.87) | 6,622.63 (3,876.50 to 10,520.50) | -3.50 (-3.84 to -3.16) | 39.74 (22.89 to 62.15) | 21.57 (12.63 to 34.26) | -1.89 (-2.10 to -1.68) |
| Mozambique | 254,424.51 (174,197.53 to 339,465.29) | 258,542.94 (150,857.80 to 399,903.13) | -2.67 (-2.82 to -2.52) | 875.32 (599.66 to 1,166.69) | 488.29 (284.90 to 755.32) | -1.83 (-1.91 to -1.76) |
| Myanmar | 289,693.45 (193,436.75 to 403,291.91 | 156,192.38 (103,460.94 to 235,124.62) | -3.10 (-3.17 to -3.02) | 542.84 (362.87 to 755.33) | 303.67 (201.15 to 457.13) | -2.04 (-2.17 to -1.92) |
| Namibia | 6,091.80 (4,175.56 to 9,153.42) | 5,041.65 (3,145.77 to 7,598.07) | -1.75 (-1.99 to -1.51) | 248.27 (170.20 to 372.93) | 183.02 (114.20 to 275.83) | -0.55 (-0.71 to -0.38) |
| Nauru | 8.13 (4.96 to 12.07) | 5.61 (3.23 to 8.99) | -1.68 (-2.27 to -1.09) | 48.08 (29.34 to 71.37) | 40.27 (23.16 to 64.51) | -0.83 (-1.37 to -0.29) |
| Nepal | 129,275.28 (82,937.58 to 194,310.19) | 53,870.52 (35,134.16 to 82,269.95) | -4.45 (-4.53 to -4.36) | 345.25 (221.79 to 518.59) | 174.93 (114.09 to 267.15 | -2.11 (-2.19 to -2.04) |
| Netherlands | 2,101.24 (1,838.02 to 2,381.30) | 1,677.08 (1,411.05 to 1,921.72) | -1.01 (-1.27 to -0.76) | 22.06 (19.29 to 25.00) | 19.54 (16.45 to 22.39) | 0.02 (-0.24 to 0.28) |
| New Zealand | 193.72 (164.02 to 224.56) | 239.56 (199.10 to 285.03) | 1.59 (0.24 to 2.96) | 6.66 (5.64 to 7.73) | 8.07 (6.71 to 9.61) | 2.78 (1.46 to 4.12) |
| Nicaragua | 21,952.15 (17,567.69 to 27,858.41) | 9,159.07 (6,428.56 to 12,653.42) | -4.20 (-4.32 to -4.08) | 321.61 (257.39 to 408.11) | 148.50 (104.24 to 205.16) | -2.43 (-2.58 to -2.28) |
| Niger | 167,404.21 (112,248.83 to 243,015.75) | 275,204.59 (164,615.72 to 423,679.96) | -2.53 (-2.76 to -2.3) | 788.19 (529.20 to 1,141.82) | 486.30 (291.07 to 748.27) | -1.88 (-2.05 to -1.71) |
| Nigeria | 1,138,682.31 (884,559.59 to 1,415,694.73) | 1,808,734.64 (1,359,994.48 to 2,350,402.86) | -1.37 (-1.6 to -1.14) | 585.58 (454.97 to 728.11) | 458.59 (344.85 to 595.79) | -0.66 (-0.76 to -0.56) |
| Niue | 0.86 (0.57 to 1.23) | 1.12 (0.75 to 1.60) | -0.81 (-1.78 to 0.16) | 38.57 (25.53 to 55.48) | 100.82 (67.56 to 144.80) | 0.20 (-0.79 to 1.2) |
| North Macedonia | 28.67 (8.93 to 57.24) | 27.37 (10.53 to 52.02) | 0.72 (-0.03 to 1.47) | 1.76 (0.55 to 3.51) | 3.00 (1.15 to 5.70) | 2.72 (1.83 to 3.61) |
| Northern Mariana Islands | 6.95 (4.40 to 10.86) | 1.43 (0.92 to 2.11) | -5.27 (-6.31 to -4.22) | 11.78 (7.45 to 18.39) | 4.98 (3.19 to 7.33) | -2.20 (-3.16 to -1.23) |
| Norway | 201.62 (181.41 to 223.48) | 50.22 (41.64 to 61.01) | -3.86 (-5.83 to -1.84) | 7.02 (6.32 to 7.78) | 1.87 (1.55 to 2.27) | -2.87 (-4.81 to -0.9) |
| Oman | 1,214.31 (741.67 to 1,863.45) | 650.95 (394.40 to 983.79) | -3.77 (-4.42 to -3.12) | 35.17 (21.48 to 53.97) | 17.04 (10.32 to 25.75) | -1.95 (-2.35 to -1.55) |
| Pakistan | 603,380.12 (433,688.87 to 817,919.17) | 789,145.68 (554,952.56 to 1,066,940.50) | -1.63 (-1.76 to -1.49) | 301.29 (216.67 to 408.58) | 265.16 (186.51 to 358.37) | -0.63 (-0.77 to -0.49) |
| Palau | 2.50 (1.41 to 3.92) | 0.98 (0.52 to 1.73) | -3.52 (-3.83 to -3.22) | 16.62 (9.34 to 26.04) | 11.29 (5.98 to 20.08) | -1.37 (-1.62 to -1.11) |
| Palestine | 2,857.90 (1,878.72 to 4,180.84) | 2,370.47 (1,541.95 to 3,430.02) | -3.25 (-3.5 to -3) | 67.61 (44.44 to 98.91) | 40.91 (26.62 to 59.18) | -1.35 (-1.5 to -1.2) |
| Panama | 3,973.55 (3,294.06 to 4,729.86) | 3,330.76 (2,565.98 to 4,279.70) | -2.07 (-2.39 to -1.76) | 139.90 (115.97 to 166.52) | 97.93 (75.43 to 125.84) | -1.00 (-1.23 to -0.76) |
| Papua New Guinea | 6,360.30 (3,554.73 to 10,052.62) | 14,751.29 (8,177.34 to 24,280.22) | -0.32 (-0.54 to -0.1) | 90.03 (50.32 to 142.30) | 90.09 (49.94 to 148.30) | -0.02 (-0.26 to 0.22) |
| Paraguay | 17,363.30 (13,345.82 to 22,078.28) | 4,404.32 (2,859.97 to 6,234.50) | -6.84 (-7.18 to -6.5) | 279.62 (214.92 to 355.56) | 70.93 (46.05 to 100.42) | -5.21 (-5.6 to -4.82) |
| Peru | 122,442.67 (86,820.28 to 165,269.56) | 53,044.45 (34,748.39 to 76,148.53) | -3.09 (-3.48 to -2.7) | 397.06 (281.46 to 535.90) | 163.78 (107.32 to 235.19) | -1.65 (-2.02 to -1.27) |
| Philippines | 352,946.16 (288,533.75 to 425,893.71) | 263,610.22 (198,741.33 to 335,662.21) | -2.45 (-2.78 to -2.11) | 364.01 (297.59 to 439.24) | 241.36 (181.96 to 307.32) | -1.07 (-1.33 to -0.81) |
| Poland | 8,471.62 (6,835.68 to 10,347.61) | 734.47 (576.07 to 929.53) | -8.65 (-9.40 to -7.90) | 32.16 (25.94 to 39.29) | 4.44 (3.49 to 5.63) | -7.92 (-8.86 to -6.97) |
| Portugal | 1,765.08 (1,476.82 to 2,036.91) | 477.48 (393.59 to 573.90) | -3.50 (-3.85 to -3.15) | 31.80 (26.60 to 36.70) | 12.04 (9.93 to 14.48) | -2.21 (-2.62 to -1.81) |
| Puerto Rico | 1,796.67 (1,569.52 to 2,039.30) | 522.31 (428.14 to 646.00) | -3.28 (-4.53 to -2.02) | 56.98 (49.77 to 64.67) | 58.93 (48.31 to 72.88) | 0.41 (-0.58 to 1.41) |
| Qatar | 40.46 (26.57 to 59.92) | 43.66 (26.51 to 64.64) | -6.09 (-6.39 to -5.78) | 7.38 (4.84 to 10.93) | 2.34 (1.42 to 3.47) | -3.67 (-3.83 to -3.51) |
| Republic of Korea | 10,967.00 (8,200.24 to 14,509.78) | 1,272.78 (830.73 to 1,753.09) | -6.92 (-7.17 to -6.67) | 33.86 (25.32 to 44.78) | 9.85 (6.43 to 13.56) | -3.68 (-4.07 to -3.29) |
| Republic of Moldova | 5,464.78 (4,383.75 to 6,508.67) | 1,863.81 (1,352.25 to 2,503.77) | -2.16 (-2.81 to -1.50) | 143.26 (114.91 to 170.64) | 136.93 (99.32 to 183.95) | -0.43 (-0.94 to 0.08) |
| Romania | 1,062.28 (736.40 to 1,351.65) | 207.02 (166.32 to 254.03) | -4.69 (-5.43 to -3.93) | 7.28 (5.04 to 9.25) | 2.40 (1.93 to 2.95) | -4.24 (-5.09 to -3.38) |
| Russian Federation | 36,558.29 (34,139.79 to 38,799.60) | 27,297.54 (24,071.28 to 30,870.48) | 0.10 (-0.47 to 0.68) | 38.29 (35.75 to 40.64) | 41.74 (36.81 to 47.21) | -0.47 (-1.1 to 0.16) |
| Rwanda | 95,114.50 (69,267.53 to 131,520.57) | 48,996.85 (31,753.95 to 69,975.97) | -3.96 (-4.26 to -3.66) | 627.11 (457.35 to 865.67) | 276.70 (179.33to 395.18) | -2.38 (-2.58 to -2.18) |
| Saint Kitts and Nevis | 16.14 (12.90 to 19.39) | 12.31 (9.23 to 16.19) | -1.80 (-2.04 to -1.56) | 36.11 (28.87 to 43.38) | 44.01 (33.00 to 57.90) | 0.99 (0.65 to 1.33) |
| Saint Lucia | 182.88 (147.74 to 222.75) | 104.13 (73.70 to 143.53) | -2.23 (-2.65 to -1.8) | 106.68 (86.17 to 129.93) | 128.58 (91.00 to 177.25) | 1.13 (0.59 to 1.67) |
| Saint Vincent and the Grenadines | 175.93 (136.01 to 223.48) | 84.87 (61.39 to 111.79) | -2.05 (-2.57 to -1.52) | 142.28 (110.00 to 180.75) | 135.15 (97.76 to 178.06) | 0.07 (-0.43 to 0.58) |
| Samoa | 73.74 (40.47 to 121.23) | 56.43 (29.29 to 88.20) | -1.51 (-1.62 to -1.39) | 28.43 (15.60 to 46.73) | 18.96 (9.84 to 29.64) | -1.22 (-1.37 to -1.07) |
| San Marino | 2.96 (1.70 to 4.76) | 0.86 (0.27 to 1.83) | -4.25 (-4.54 to -3.95) | 25.12 (14.46 to 40.46) | 7.72 (2.47 to 16.47) | -2.93 (-3.22 to -2.64) |
| Sao Tome and Principe | 835.98 (549.27 to 1,256.10) | 420.00 (252.31 to 701.44) | -4.03 (-4.52 to -3.54) | 377.48 (248.06 to 566.98) | 177.03 (106.31 to 295.65) | -2.44 (-2.65 to -2.22) |
| Saudi Arabia | 22,276.39 (12,525.75 to 35,825.39) | 4,290.33 (2,351.52 to 6,710.95) | -8.06 (-8.44 to -7.67) | 90.74 (51.03 to 145.93) | 19.15 (10.49 to 29.99) | -5.36 (-5.76 to -4.95) |
| Senegal | 110,528.73 (77,012.71 to 152,843.10) | 88,429.14 (61,957.91 to 122,334.36) | -2.64 (-2.8 to -2.47) | 670.31 (467.24 to 926.34) | 383.72 (268.88 to 530.87) | -1.54 (-1.65 to -1.44) |
| Serbia | 1,273.10 (692.70 to 2,187.27) | 203.29 (135.91 to 279.06) | -6.76 (-7.41 to -6.1) | 19.23 (10.46 to 33.05) | 6.27 (4.19 to 8.60) | -4.73 (-5.37 to -4.08) |
| Seychelles | 105.49 (72.23 to 152.42) | 78.04 (48.18 to 115.65) | -1.65 (-1.89 to -1.41) | 133.19 (91.19 to 192.49) | 102.60 (63.35 to 152.09) | -0.57 (-0.7 to -0.45) |
| Sierra Leone | 86,933.57 (54,857.03 to 125,831.97) | 76,171.72 (50,444.00 to 110,013.60) | -2.65 (-2.99 to -2.31) | 900.04 (568.28 to 1,300.32) | 527.51 (349.49 to 761.56) | -1.59 (-1.85 to -1.34) |
| Singapore | 97.67 (83.35 to 113.31) | 61.06 (47.67 to 76.54) | -3.54 (-3.98 to -3.1) | 4.02 (3.43 to 4.66) | 2.29 (1.79 to 2.87) | -1.78 (-2.14 to -1.43) |
| Slovakia | 85.45 (57.65 to 121.27) | 48.17 (33.30 to 66.97) | -1.53 (-1.98 to -1.09) | 2.23 (1.51 to 3.17) | 1.79 (1.24 to 2.49) | -0.90 (-1.56 to -0.24) |
| Slovenia | 6.88 (3.92 to 10.27) | 7.70 (4.82 to 11.23) | 1.23 (-0.08 to 2.57) | 0.64 (0.36 to 0.95) | 0.85 (0.53 to 1.25) | 1.23 (-0.12 to 2.59) |
| Solomon Islands | 544.65 (318.32 to 887.85) | 586.20 (348.39 to 994.00) | -2.06 (-2.32 to -1.79) | 83.20 (48.63 to 135.63) | 59.88 (35.57 to 101.55) | -1.03 (-1.23 to -0.83) |
| Somalia | 113,872.51 (62,022.58 to 169,611.06) | 248,258.73 (123,732.70 to 402,009.63) | -0.57 (-0.77 to -0.37) | 618.03 (336.52 to 920.12) | 540.38 (269.43 to 874.44) | -0.34 (-0.45 to -0.23) |
| South Africa | 94,898.67 (72,268.82 to 119,325.35) | 89,911.05 (69,460.84 to 111,160.11) | -1.15 (-1.42 to -0.89) | 189.12 (144.03 to 237.81) | 190.22 (146.96 to 235.18) | 0.05 (-0.11 to 0.22) |
| South Sudan | 68,622.39 (41,343.91 to 102,423.78) | 85,743.34 (43,130.11 to 154,147.54) | -0.79 (-1.02 to -0.56) | 562.47 (338.30 to 839.75) | 470.08 (236.57 to 844.27) | -0.36 (-0.49 to -0.24) |
| Spain | 4,651.10 (4,141.62 to 5,229.65) | 2,496.03 (2,105.62 to 2,927.18) | -1.59 (-2.24 to -0.93) | 24.18 (21.53 to 27.20) | 15.40 (12.99 to 18.06) | -0.96 (-1.33 to -0.59) |
| Sri Lanka | 24,293.55 (18,629.76 to 31,534.14) | 7,654.35 (5,364.62 to 10,714.61) | -4.37 (-4.74 to -3.99) | 140.62 (107.84 to 182.54) | 52.60 (36.86 to 73.68) | -3.30 (-3.58 to -3.02) |
| Sudan | 22,920.83 (13,845.65 to 36,128.50) | 18,303.46 (10,749.56 to 28,795.67) | -3.08 (-3.29 to -2.87) | 55.12 (33.27 to 86.92) | 33.21 (19.50 to 52.25) | -1.61 (-1.66 to -1.55) |
| Suriname | 1,179.16 (843.92 to 1,666.92) | 946.59 (608.01 to 1,361.86) | -1.68 (-1.85 to -1.5) | 268.64 (192.28 to 379.78) | 221.66 (142.35 to 318.89) | -0.42 (-0.59 to -0.24) |
| Sweden | 690.13 (597.43 to 796.23) | 290.26 (241.09 to 346.79) | -2.69 (-3.37 to -2) | 11.54 (9.99 to 13.32) | 5.27 (4.38 to 6.30) | -2.47 (-3.15 to -1.77) |
| Switzerland | 501.05 (430.50 to 578.00) | 460.24 (363.43 to 570.01) | -1.20 (-1.47 to -0.92) | 12.36 (10.62 to 14.26) | 10.85 (8.57 to 13.44) | -0.62 (-0.89 to -0.35) |
| Syrian Arab Republic | 11,913.29 (6,908.64 to 20,120.97) | 1,678.35 (810.24 to 3,238.79) | -5.97 (-6.31 to -5.63) | 54.08 (31.36 to 91.34) | 17.71 (8.55 to 34.20) | -3.02 (-3.29 to -2.75) |
| Taiwan (Province of China) | 152.28 (128.83 to 177.89) | 1,162.53 (965.67 to 1,407.86) | 7.50 (6.63 to 8.39) | 0.97 (0.82 to 1.13) | 15.28 (12.70 to 18.51) | 10.55 (9.57 to 11.53) |
| Tajikistan | 4,817.82 (2,513.41 to 8,227.55) | 5,501.18 (2,893.16 to 9,658.72) | -1.28 (-1.4 to -1.15) | 48.20 (25.15 to 82.31) | 40.44 (21.27 to 71.00) | -0.63 (-0.84 to -0.42) |
| Thailand | 80,416.14 (48,885.19 to 127,124.43) | 12,841.06 (9,764.06 to 16,337.40) | -6.01 (-6.27 to -5.74) | 161.14 (97.95 to 254.74) | 49.54 (37.66 to 63.06) | -3.53 (-3.76 to -3.29) |
| Timor-Leste | 6,384.36 (4,084.79 to 9,049.11) | 4,132.17 (2,808.28 to 5,905.72) | -3.80 (-4.04 to -3.57) | 383.94 (245.74 to 543.80) | 210.95 (143.36 to 301.49) | -2.24 (-2.4 to -2.08) |
| Togo | 46,448.94 (32,197.21 to 66,744.48) | 39,687.33 (25,998.46 to 58,299.42) | -2.82 (-3.03 to -2.60) | 606.54 (420.95 to 870.99) | 341.25 (223.59 to 501.28) | -1.74 (-1.81 to -1.67) |
| Tokelau | 1.12 (0.54 to 1.99) | 2.27 (0.77 to 4.05) | -1.68 (-3.27 to -0.06) | 63.86 (30.89 to 113.15) | 265.99 (90.23 to 475.48) | 0.04 (-1.61 to 1.71) |
| Tonga | 64.31 (33.65 to 105.97) | 39.00 (18.85 to 67.36) | -1.62 (-1.86 to -1.37) | 41.11 (21.50 to 67.80) | 27.23 (13.18 to 47.08) | -1.23 (-1.48 to -0.99) |
| Trinidad and Tobago | 2,146.29 (1,703.98 to 2,615.28) | 1,254.59 (929.85 to 1,703.13) | -1.54 (-1.82 to -1.25) | 186.86 (148.35 to 227.70) | 175.32 (129.94 to 238.02) | -0.30 (-0.56 to -0.05) |
| Tunisia | 2,469.33 (1,364.40 to 4,048.57) | 972.31 (503.45 to 1,909.63) | -3.27 (-3.52 to -3.03) | 23.47 (12.96 to 38.49) | 12.07 (6.26 to 23.70) | -1.92 (-2.02 to -1.83) |
| Turkey | 113,756.07 (77,358.96 to 159,097.30) | 25,986.69 (16,157.07 to 38,010.34) | -5.56 (-5.7 to -5.41) | 160.55 (109.19 to 224.57) | 53.39 (33.17 to 78.13) | -3.55 (-3.69 to -3.42) |
| Turkmenistan | 2,200.82 (1,559.10 to 3,040.90) | 1,977.87 (1,404.44 to 2,721.52) | -0.72 (-1.01 to -0.43) | 36.59 (25.93 to 50.56) | 37.31 (26.49 to 51.34) | 0.59 (0.31 to 0.87) |
| Tuvalu | 7.08 (3.52 to 13.20) | 3.14 (1.73 to 5.57) | -3.42 (-3.5 to -3.33) | 43.08 (21.40 to 80.32) | 24.53 (13.52 to 43.48) | -1.84 (-1.98 to -1.7) |
| Uganda | 216,377.52 (155,745.32 to 296,640.64) | 266,157.49 (184,187.75 to 373,456.34) | -2.16 (-2.39 to -1.93) | 491.92 (354.18 to 673.59) | 352.13 (243.72 to 494.10) | -0.95 (-1.09 to -0.81) |
| Ukraine | 10,104.58 (7,335.83 to 13,506.80) | 4,030.37 (3,067.50 to 5,091.40) | -0.69 (-1.23 to -0.15) | 31.27 (22.70 to 41.81) | 30.38 (23.13 to 38.39) | 0.15 (-0.06 to 0.36) |
| United Arab Emirates | 128.75 (53.72 to 299.58) | 115.77 (65.27 to 238.83) | -3.77 (-4.34 to -3.2) | 5.53 (2.31 to 12.88) | 3.15 (1.77 to 6.49) | -0.57 (-0.97 to -0.17) |
| United Kingdom | 2,973.61 (2,549.21 to 3,349.60) | 962.72 (777.48 to 1,149.36) | -4.34 (-4.75 to -3.93) | 7.75 (6.64 to 8.73) | 2.87 (2.31 to 3.42) | -3.80 (-4.26 to -3.35) |
| United Republic of Tanzania | 304,036.37 (209,294.73 to 424,298.30) | 393,097.66 (256,203.98 to 606,155.28) | -1.28 (-1.49 to -1.08) | 529.33 (364.41 to 737.82) | 435.46 (283.81 to 671.49) | -0.16 (-0.35 to 0.03) |
| United States of America | 33,664.22 (31,243.06 to 36,171.90) | 27,471.05 (24,327.55 to 30,565.68) | -1.33 (-1.73 to -0.94) | 16.77 (15.56 to 18.03) | 15.44 (13.67 to 17.18) | -0.24 (-0.57 to 0.09) |
| United States Virgin Islands | 44.79 (31.22 to 63.45) | 9.59 (5.26 to 15.13) | -3.71 (-3.84 to -3.59) | 40.15 (27.98 to 56.89) | 27.37 (15.01 to 43.15) | -0.66 (-0.81 to -0.51) |
| Uruguay | 2,756.49 (2,367.03 to 3,118.49) | 805.17 (632.45 to 1,048.11) | -4.44 (-4.78 to -4.09) | 102.43 (87.95 to 115.89) | 46.96 (36.89 to 61.15) | -2.95 (-3.31 to -2.58) |
| Uzbekistan | 10,379.86 (7,528.17 to 15,696.91) | 23,967.80 (19,149.28 to 29,148.87) | 1.97 (1.48 to 2.46) | 30.45 (22.08 to 46.04) | 62.52 (49.95 to 76.05) | 2.79 (2.30 to 3.27) |
| Vanuatu | 153.96 (85.81 to 246.80) | 174.44 (101.52 to 282.95) | -2.01 (-2.39 to -1.62) | 52.74 (29.39 to 84.55) | 41.46 (24.13 to 67.29) | -0.89 (-1.23 to -0.54) |
| Venezuela (Bolivarian Republic of) | 47,051.40 (40,095.03 to 54,995.46) | 28,372.79 (19,511.58 to 40,987.74) | -1.96 (-2.31 to -1.61) | 178.11 (151.77 to 208.19) | 130.56 (89.77 to 188.61) | -0.71 (-1.02 to -0.4) |
| Viet Nam | 214,436.00 (143,293.25 to 308,432.46) | 58,863.78 (38,260.12 to 89,557.95) | -5.13 (-5.35 to -4.92) | 230.97 (154.37 to 332.16) | 78.78 (51.20 to 119.92) | -3.78 (-3.9 to -3.65) |
| Yemen | 15,946.73 (9,779.06 to 24,876.49) | 17,620.17 (10,271.61 to 26,733.76) | -2.27 (-2.56 to -1.98) | 50.95 (31.24 to 79.52) | 37.84 (22.06 to 57.41) | -0.84 (-0.91 to -0.78) |
| Zambia | 81,454.41 (57,406.18 to 110,813.50) | 100,109.64 (65,095.56 to 147,022.95) | -1.92 (-2.32 to -1.52) | 446.46 (314.74 to 606.99) | 345.70 (224.80 to 507.68) | -0.66 (-0.87 to -0.45) |
| Zimbabwe | 43,013.70 (29,828.44 to 61,109.00) | 59,231.35 (42,497.51 to 84,992.70) | 0.76 (0.33 to 1.19) | 231.69 (160.70 to 329.09) | 264.70 (189.96 to 379.72) | 1.17 (0.87 to 1.47) |

DALYs, disability-adjusted life years; ASDR, age-standardized DALYs rate; EAPC, estimated annual percentage change; UI, uncertainty interval; CI, confidence interval.

Supplementary Table S3.

Predicted age-standardized rate of deaths and disability-adjusted life years (DALYs) per 100,000 for neonatal sepsis and other neonatal infections attributable to low birth weight, 2022 - 2031.

| **measure** | **year** | **mean** | **low** | **up** |
| --- | --- | --- | --- | --- |
| Deaths | 2022 | 2.19 | 2.16 | 2.22 |
|  | 2023 | 2.16 | 2.10 | 2.23 |
|  | 2024 | 2.14 | 2.03 | 2.24 |
|  | 2025 | 2.11 | 1.95 | 2.27 |
|  | 2026 | 2.08 | 1.87 | 2.30 |
|  | 2027 | 2.06 | 1.78 | 2.34 |
|  | 2028 | 2.03 | 1.69 | 2.38 |
|  | 2029 | 2.01 | 1.59 | 2.43 |
|  | 2030 | 1.98 | 1.49 | 2.48 |
|  | 2031 | 1.96 | 1.38 | 2.53 |
| DALYs | 2022 | 196.65 | 194.02 | 199.28 |
|  | 2023 | 194.37 | 188.49 | 200.24 |
|  | 2024 | 192.08 | 182.25 | 201.91 |
|  | 2025 | 189.79 | 175.40 | 204.19 |
|  | 2026 | 187.51 | 168.02 | 206.10 |
|  | 2027 | 185.22 | 160.16 | 210.29 |
|  | 2028 | 182.94 | 151.85 | 214.03 |
|  | 2029 | 180.65 | 143.12 | 218.18 |
|  | 2030 | 178.37 | 134.01 | 222.73 |
|  | 2031 | 176.08 | 124.53 | 227.64 |
